# Superluminal light propagation in a three-level ladder system

**DOI:** 10.1038/s41598-024-62220-x

**Published:** 2024-07-02

**Authors:** Piotr Gładysz, Szymon Pustelny, Karolina Słowik

**Affiliations:** 1 Institute of Physics, Faculty of Physics, Astronomy and Informatics, Nicolaus Copernicus University in Toruń, Grudziadzka 5, 87-100 Toruń, Poland; 2 Institute of Physics, Jagiellonian University, Łojasiewicza 11, 30-348 Kraków, Poland

**Keywords:** Atom optics, Nonlinear optics

## Abstract

Superluminal light propagation is typically accompanied by significant absorption that might prevent its observation in realistic samples. We propose an all-optical implementation exploiting the two-photon resonance in three-level media to overcome this problem. With several computational methods, we analyze three possible configurations of optically-dressed systems and identify an optimal configuration for superluminal propagation. Due to the far-detuned operating regime with low absorption, this scenario avoids the usual need for population inversion, gain assistance or nonlinear optical response. Our analysis covers a broad parameter space and aims for the identification of conditions where significant pulse advancement can be achieved at high transmission levels. In this context, a figure of merit is introduced accounting for a trade-off between the desired group-index values and transmission level. This quantity helps to identify the optimal characteristics of the dressing beam.

## Introduction

**Figure 1:**
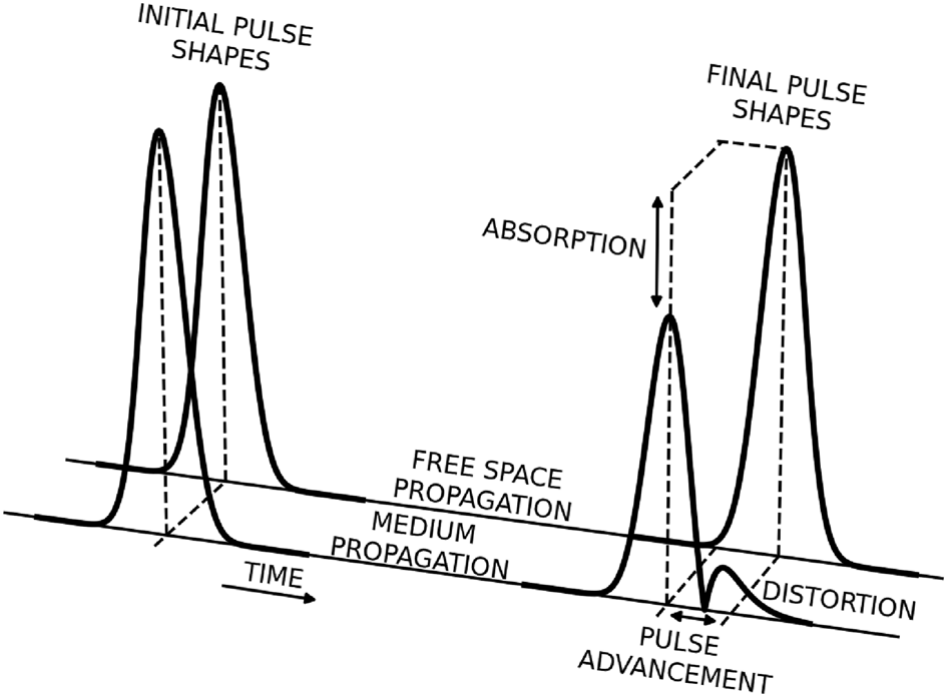
Schematic representation of the pulse propagating in the time domain through the free space (behind) and through the medium (in the front) of equal lengths. On the left-hand side, we present two identical Gaussian pulses (for z=0), while on the right-hand side, we show an unchanged shape in free propagation and a distorted one for the medium case (for z=L).

Throughout the decades, the perspective of propagation of electromagnetic pulses with velocities greater than the speed of light in vacuum *c* has inspired the imagination of researchers. Theoretical predictions and experimental evidence of superluminal propagation were subjects of wide-ranging discussions, in particular in the context of Einstein’s special relativity^1–3^. As a result of these debates and numerous follow-up works, a number of scenarios^4^ for slow (subluminal)^5–7^, fast (superluminal)^8–12^, or tunable propagation velocity^13–17^ were implemented. With this work, we propose a new, simple experimental scheme for superluminal propagation, adding to this extensive topic. The schematic superluminal pulse propagation in the time domain is shown in Fig. 1. The pulse propagating through the medium arrives faster (pulse advancement) and is also distorted and partially absorbed compared to the pulse propagating through the free space of the same length.

The group index describes the medium’s optical properties and is linked to its dispersion. Specifically, normal dispersion results in subluminal propagation with $0\le v_g\le c$.^18,19^ Anomalous dispersion can lead to pulse velocities exceeding the vacuum speed of light $v_g>c$,^20,21^ or negative $v_g<0$.^11^ The two latter cases are called superluminal light propagation. The terms *slow* and *fast* light can be described by the group refractive index $n_g$ related to the pulse group velocity via $v_g=c/n_g$.^22^

Anomalous dispersion is a requirement for superluminal pulse propagation, but its observation in realistically long samples is often hindered by the associated high absorption^23–26^. In some experiments researchers address this issue^27,28^, however, in general, this leads to distorted pulses and challenges in defining group velocity^29–31^. To overcome this problem, several schemes based on electromagnetically induced transparency (EIT)^32–35^, optical saturation^36^, gain assistance^8,10,37^, four-wave mixing^38^, and many more^12,15,39^ have been proposed.

This work demonstrates that superluminal pulse propagation could be achieved in a significantly simpler scenario. It involves a coherent interaction between an atomic medium and two light beams (control and probe), modifying the medium’s optical properties and the probe-pulse group velocity via the control field. In the proposed scheme, the probe and control fields are far detuned from single-photon transitions and are tuned near a two-photon transition, reducing absorption and limiting population transfer and pulse distortion. Under such conditions, the anomalous dispersion of the medium can be exploited without gain assistance and far from saturation. The predicted levels of pulse advancement are comparable to those achieved in gain-assisted schemes^9,40^ for similarly high transmittance and low pulse distortion.

In the manuscript, we examine different energy-level schemes in a three-level system for superluminal pulse propagation and identify the ladder scheme as a good candidate, offering a small group index and low absorption. This scheme is then theoretically investigated in a broad range of control parameters to identify regimes where superluminal propagation with considerable pulse advancement can be achieved at high transmission levels with little pulse distortion. Several approaches are used to determine the group index of the medium. The approaches exploit elegant but approximate analytical methods, but also exact numerical treatment. While the analytical method works for monochromatic waves, the determination of the group velocity of a pulse requires analysis of its whole spectrum, which typically calls for numerical analysis. These approaches are used for the analysis of pulse velocity in rubidium vapors dressed with the control field. We highlight the trade-off between the negative group velocity of a pulse and its transmission coefficient. To quantify this trade-off, we introduce a figure of merit and use it to identify the optimal control-field parameters for the superluminal propagation of the probe.

## Model

**Figure 2:**
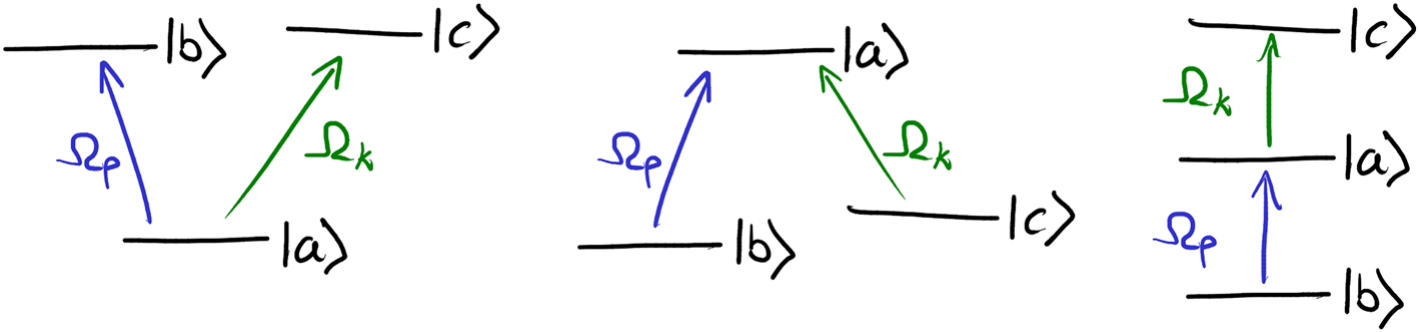
Investigated three-level configurations coupled by the probe (blue) and control (green) fields of Rabi frequencies $\Omega _p$ and $\Omega _k$.

A two-level system is the simplest system with anomalous dispersion^41^, but the associated strong on-resonance absorption limits its usefulness for the investigation of superluminal propagation. Therefore, we explore three-level media in the so-called vee (*V*), lambda ($\Lambda$), and ladder ($\Xi$) configurations (Fig. 2). In each scheme, selected pairs of levels are coupled with the probe $\Omega _p$ and control $\Omega _k$ fields. This allows controlling probe propagation with the control field. The medium is described using the density matrix $\rho$, whose evolution is governed by the Bloch equations. The matrix can be used to determine the medium’s polarization at any time and position along the propagation direction, being the source term for the coupled first-order field propagation equation.

A derivation of the Bloch-Maxwell equations for one-dimensional media for the three cases is provided in Supplement Sec. S1. The resulting Bloch evolution equations have the form

1a $$\begin{aligned} \dot{\sigma }_{aa}&=\textrm{i}\Omega _p\sigma _{ba}-\textrm{i}\Omega _p\sigma _{ab}-\textrm{i}\Omega _k\sigma _{ac}+\textrm{i}\Omega _k\sigma _{ca}+\mathscr {L}_{aa}, \end{aligned}$$

1b $$\begin{aligned} \dot{\sigma }_{cc}&=\textrm{i}\Omega _k\sigma _{ac}-\textrm{i}\Omega _k\sigma _{ca}+\mathscr {L}_{cc},\end{aligned}$$

1c $$\begin{aligned} \dot{\sigma }_{ba}&=\alpha _p\textrm{i}\delta _p\sigma _{ba}-\textrm{i}\Omega _p(1-\sigma _{cc}-2\sigma _{aa})-\textrm{i}\Omega _k\sigma _{bc}+\mathscr {L}_{ba},\end{aligned}$$

1d $$\begin{aligned} \dot{\sigma }_{ac}&=-\alpha _k\textrm{i}\delta _k\sigma _{ac}+\textrm{i}\Omega _p\sigma _{bc}-\textrm{i}\Omega _k(\sigma _{aa}-\sigma _{cc})+\mathscr {L}_{ac},\end{aligned}$$

1e $$\begin{aligned} \dot{\sigma }_{bc}&=(\alpha _p\textrm{i}\delta _p-\alpha _k\textrm{i}\delta _k)\sigma _{bc}+\textrm{i}\Omega _p\sigma _{ac}-\textrm{i}\Omega _k\sigma _{ba}+\mathscr {L}_{bc}, \end{aligned}$$

where $\sigma$ is the density matrix in the rotating frame, $\delta _{p,k}$ are detunings of the fields from resonances, and $\mathscr {L}$ is a decoherence term (see Supplement Sec. S2).

The equations describing the field propagation in the slowly-varying-envelope approximation read

2 $$\begin{aligned} \left( \partial _t\pm c\partial _z\right) \Omega _{p/k}=\pm \alpha _{p/k}\textrm{i}\frac{N\omega _{p/k}\left| d_{ba/ac} \right| ^2}{2\hbar \epsilon _0}\sigma _{ba/ac}, \end{aligned}$$

where the left/right indices correspond to the probe/control fields *N* is the atomic concentration, $\omega _{p/k}$ are fields central frequencies, $d_{ba/ac}$ are values of the transition dipole moments, and $\epsilon _0$ is electric permittivity in vacuum. The ± signs allow us to choose the propagation direction: minus for propagation in the direction of the axis and plus otherwise. We have introduced parameters $\alpha _{p/k}$ to keep track of the signs as follows: for the *V* system: $\alpha _p=\alpha _k=+1$, for the $\Lambda$ system: $\alpha _p=\alpha _k=-1$, for the $\Xi$ system: $\alpha _p=-1$, $\alpha _k=+1$. All the quantities are described in greater detail in Supplement Sec. S1.

## Identification of the optimal three-level system

**Figure 3:**
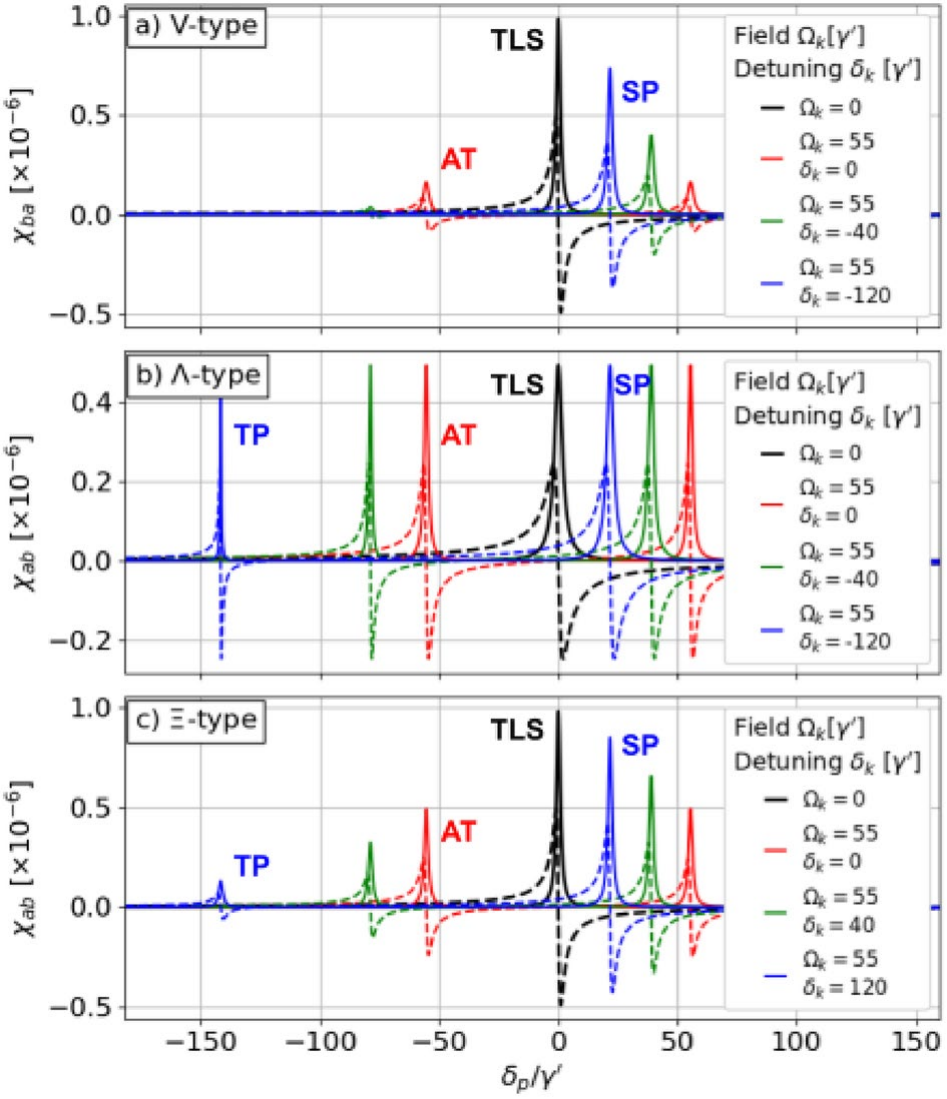
Real (dashed) and imaginary (solid) parts of the electric susceptibility as functions of the probe detuning for different energy-level schemes. The black curves illustrate the single-photon resonance in the absence of the control field (TLS regime), while other colors illustrate results for $\Omega _k=\gamma ^\prime$ and different $\delta _k$s (AT, SP, TP regimes). The control field splits the single resonance (black) into two resonances (colors). For a) and b), the two-photon resonance condition reads $\delta +\delta _k = 0$ while for c) it is $\delta - \delta _k = 0$. Note that each absorption peak (solid lines) is accompanied by anomalous dispersion (dashed lines).

The purpose of this section is to identify the appropriate configuration to achieve superluminal propagation. Based on investigations of the electric susceptibility $\chi$, we seek a balance between the adverse strength of absorption and the favorable slope of dispersion. Under such balanced conditions, the pulse propagates with a superluminal velocity and maintains its temporal shape.

### Three-level systems

The electric susceptibilities $\chi _{ab/ba}$ for the three configurations are derived in Supplement Sec. S4 with a description of approximations and assumptions, and presented in the main text in Fig. 3 for selected parameters’ values. The susceptibility functions sustain resonances whose nature is described in detail in the following paragraphs. Each of these resonances supports anomalous dispersion (dashed lines) being the necessary condition for superluminality. By Kramers-Krönig relations, the anomalous dispersion is in each case fundamentally linked to the accompanying absorption peak (solid lines)^2^. In the absence of the control field, the susceptibility sustains a single resonance for each of the three configurations (black lines in Fig. 3). The resonance corresponds to the transition between the levels $\vert a \rangle$ and $\vert b \rangle$ , while the level $\vert c \rangle$ contributes little to the dynamics leading to a two-level system (TLS) regime. A relatively strong, near-resonant control field splits the resonance into a pair of symmetric resonances (red lines). This splitting is a consequence of the Autler-Townes (AT) or EIT effects^42^. A more interesting situation arises when the control field is significantly detuned from the single-photon transition between the $\vert a \rangle$ and $\vert c \rangle$ levels ($|\delta _k|\gg \gamma _{cc}$, $\gamma _{aa}$). With the negatively-detuned control field, $\delta _k<0$ in *V* and $\Lambda$ cases, and positively-detuned $\delta _k>0$ in $\Xi$ case, the resonances that arise at positive probe detunings ($\delta >0$; right peaks in the plots) can be associated with single-photon transitions $\vert b \rangle \rightarrow \vert a \rangle$ (eg. blue curves, single-photon (SP) regime) while the resonances that arise at negative probe detunings (left peaks in the plots) - as a consequence of two-photon transitions $\vert b \rangle \rightarrow \vert c \rangle$ (eg. blue curves, two-photon (TP) regime). This classification reflects the appearance of $\textrm{i}\delta$ (single-photon transition), and $\textrm{i}(\delta \pm \delta _k)$ (two-photon transition) terms in expressions for electric susceptibilities (see Eqs. S24 in the Supplement). As the value of the control-field detuning $|\delta _k|$ increases, the single-photon resonance moves closer to the single-photon resonance obtained for $\Omega _k = 0$. This indicates that the system is weaker perturbed by the control field. The behaviour of the two-photon resonance strongly depends on the scheme of energy levels. The two-photon resonance amplitude and width are related to the decoherence rate $\gamma _{bc}$ between the $\vert b \rangle$ and $\vert c \rangle$ levels. As derived in the Supplement Sec. S4, in the considered case $\gamma _{bc}$ is given by $\frac{1}{2}(\gamma _{bb}+\gamma _{cc})$, 0, and $\frac{1}{2}\gamma _{cc}$ for the *V*, $\Lambda$, and $\Xi$ configuration, respectively. This causes the different scaling of the resonance peaks we discuss now.

In the *V* system (Fig. 3a) with two short-living levels, a significant population transfer occurs from the ground level $\vert a \rangle$ to the excited state $\vert c \rangle$ due to the illumination with the strong control field $\left( \sigma _{aa} = \frac{\gamma ^2_{cc} + 4(\delta ^2_k + \Omega ^2_k)}{\gamma ^2_{cc} + 4(\delta ^2_k + 2\Omega ^2_k)} + O[\Omega ^2_p] \right)$. Such population distribution makes the system more vulnerable to decoherence^42^. Due to the relatively large decoherence rate $\gamma _{bc}$, the two-photon resonance amplitude decreases rapidly with the control-field detuning.

Due to the existence of two infinitely long-lived levels $\vert b \rangle$ and $\vert c \rangle$, the two-photon resonance is narrowest in the $\Lambda$ system (Fig. 3b). The amplitude of the resonance is comparable to the single-photon resonance, and it does not depend on the detuning. In turn, strong two-photon absorption is observed. In practical implementations, the lifetime of the two lower-energy levels is limited by atomic collisions or the exchange of atoms with a reservoir. Consequently, under realistic conditions, the two-level absorption is suppressed compared to the single-photon absorption, decreasing with the detuning.

The $\Xi$ system (Fig. 3c) has advantages over both *V* and $\Lambda$ systems: The transfer of population $(\sigma _{bb} = 1 + O[\Omega ^2_p])$ and the amplitude of the two-photon resonance are relatively small, corresponding to suppressed absorption. At the same time, the two-photon resonance width is narrow leading to a relatively steep derivative and, hence, large group velocities. This makes the $\Xi$-type system, driven by a weak probe field and strong, far-detuned control field particularly attractive for superluminal pulse propagation. Contrary to the intensively studied $\Lambda$ configuration, the system has not been studied in detail in this context, so hereafter we focus our analysis on the $\Xi$ system. Its electric susceptibility reads

3 $$\begin{aligned} \chi _{ab}(\delta )=\frac{N\left| d_{ab} \right| ^2}{\hbar \epsilon _0}\frac{\textrm{i}}{-\textrm{i}\delta +\frac{\gamma _{aa}}{2}+\frac{\Omega ^2_k}{-\textrm{i}(\delta +\delta _k)+\frac{\gamma _{cc}}{2}}}+\mathcal {O}[\Omega ^2_p], \end{aligned}$$

where $\delta$ plays the role of the probe detuning.

### Relation to two-level media

Similar conditions can be achieved in a low-density two-level medium characterized with a single-photon resonance. However, such resonance is typically relatively strong resulting in full pulse absorption even for low medium densities. $\Xi$-type systems provide a more realistic platform for experiments as densities can be higher and fields can be stronger without inducing significant population transfer. We can analyze it more precisely in terms of saturation parameters in both cases. A large saturation parameter means strong population transfer that leads to pulse distortion.

For the two-level system, the Hamiltonian in the interaction picture reads

4 $$\begin{aligned} H\dot{=}\frac{\hbar }{2}\begin{bmatrix} \delta_{TLS} &{} -\Omega \\ -\Omega ^* &{} -\delta_{TLS} \end{bmatrix}, \end{aligned}$$

where $\delta_{TLS}$ is the detuning from the resonance and $\Omega$ is Rabi frequency. Based on this, we can simply write saturation parameter for some fixed spontaneous emission rate $\gamma$

5 $$\begin{aligned} S=\frac{2\left| \Omega \right| ^2}{1+\left( \frac{2\delta_{TLS} }{\gamma }\right) ^2}. \end{aligned}$$

This parameter directly corresponds to the population of the excited level in the steady state given by $\frac{S/2}{1+S}$.

To compare this result with our three-level ladder model we can perform adiabatic elimination of the middle level^43^. This can be done as the level is approximately empty due to the assumed huge values of single-photon detuning. We obtain an effective two-level Hamiltonian

6 $$\begin{aligned} H^{\Xi }_\text {eff}\dot{=}\frac{\hbar }{2}\begin{bmatrix} (\delta _p+\delta _k)-\frac{\left| \Omega _k \right| ^2}{\delta _p-\delta _k} &{} -\frac{\Omega _p\Omega _k}{\delta _p-\delta _k}\\ -\frac{\Omega ^*_p\Omega ^*_k}{\delta _p-\delta _k}&{} -(\delta _p+\delta _k)-\frac{\left| \Omega _p \right| ^2}{\delta _p-\delta _k} \end{bmatrix}, \end{aligned}$$

In analogy, we can write the effective saturation parameter for the ladder system. Let us assume comparable values of the spontaneous emission rates in both models $\gamma ^\Xi \approx \gamma$, comparable fields values $\left| \Omega _p \right| \approx \left| \Omega _k \right| \approx \left| \Omega \right|$ and introducing $\delta_{TLS} =\delta _p+\delta _k$ as a two-photon detuning and $\Delta =\delta _p-\delta _k\approx -2\delta _k$ being of the order of the single-photon detuning. Since $\Delta \gg \delta_{TLS}$ we can neglect $\left| \Omega \right| ^2/\Delta$ term and by comparison to the two-level description (Eqs. (4, 5) with Eq. (6)), express the resulting effective saturation parameter $S^\Xi$ with respect to the *S* parameter

7 $$\begin{aligned} S^{\Xi }\approx \frac{2\left| \Omega \right| ^4}{\Delta ^2\left( 1+\left( \frac{2\delta_{TLS} }{\gamma }\right) ^2\right) }=\frac{\left| \Omega \right| ^2}{\Delta ^2}S \ll S. \end{aligned}$$

Thus, we rather qualitatively show the intuitive result that the saturation in the ladder-type systems compared to the pure two-level one is much harder to achieve hence the population transfer is suppressed by the factor $\frac{\left| \Omega \right| ^2}{\Delta ^2}$ which is roughly of the order of $10^{-2}-10^{-4}$. Indeed, experiments in three-level media are usually carried out far from the saturation regime^13,25^.

## Group index: approaches

The group velocity $v_g$ and the group index $n_g$ are related by the expression $v_g=c/n_g$. This paper mainly examines the group index, which is more convenient for calculations. The following section describes different approaches enabling the determination of the group index in optically-dressed media. The analytical derivation of the group index is based on an assumption of the monochromatic probe light. For a more realistic treatment, we additionally consider numerical and semi-analytical methods to incorporate the spectral shape of the probe pulse.

### Analytical solution

The spectral components of the group index can be related to the electric susceptibility by^44^

8 $$\begin{aligned} n_g(\delta )=\Re \left( \sqrt{1+\chi _{ab}(\delta )} \right) +\omega _p \frac{\partial }{\partial \delta }\Re \left( \sqrt{1+\chi _{ab}(\delta )} \right) . \end{aligned}$$

The electric susceptibility of the $\Xi$-type medium is given by Eq. (3), which holds for $\Omega _k\gg \Omega _p$. For a more detailed discussion including nonlinear probe corrections see Supplement Sec. S5.1. The analytical formulation in (8) assumes spectrally narrow pulses; hence, we will use it as a benchmark for more realistic calculations considering spectrally broad pulses.

### Numerical approach

The numerical method operates entirely in the time domain. It achieves the best simulation accuracy at the expense of the highest computational time among all the considered methods. We have implemented Eqs. (1) and (2) in a self-developed solver in Python based on the Lax-Wendroff and Runge-Kutta 4th order methods (see Supplement Sec. S3). It provides the full dynamics for both medium and fields conforming to the slovly-varying-envelope approximation.

Knowing the pulse’s temporal profile at the end of the sample $\Omega _p(z=L,t)$, we calculate the group index in analogy to the optical refractive index, evaluating the difference in optical (here “group”) paths for pulses propagating inside the medium and in free space: $n_g L - L = ct_g - ct_0$. Here, $t_g\equiv t_g(\delta _p, \Omega _k, L)$ is the time of travel of a fixed-carrier-frequency probe pulse through the given medium, while $t_0$ is the time for the same pulse travelling through empty space of the same length *L*. This yields^45^

9 $$\begin{aligned} n_g(\delta _p)=1+\frac{c}{L}(t_g(\delta _p)-t_0). \end{aligned}$$

Evaluation of $t_g$ has to be based on a reference point in the pulse, which we select as the maximum of the pulse. In optically-dense media the shape of a pulse may be distorted (see schematics shown in Fig. 1), which calls into question our ability to track the pulse maximum, and hence, the definition of group velocity^44,46^. In our study, we use Gaussian pulses in dilute media to ensure the preservation of pulse shape and carefully monitor population distribution and absorption rate.

The expression given by Eq. (8) describes individual spectral components of the group index. Contrary, (Eq. 9) is a recipe to evaluate the group index of a pulse with the carrier frequency detuning $\delta _p$. The pulse could be spectrally broad as demonstrated in Sec. 5 and might have a nontrivial shape, with the restrictions described in the previous paragraph.

### Fourier semi-analytical solution

The final group index evaluation method combines elements from frequency and time domains and ideas already presented. We Fourier transform (FT) the propagation equation for the probe field [Eq. (2)] and by using relation $\tilde{\sigma }_{ab} \propto \tilde{\Omega }_p\chi _{ab}$ (see Supplement) and (Eq. 3), we obtain

10 $$\begin{aligned} \left( \frac{\omega }{c}-\textrm{i}\partial _z\right) \tilde{\Omega }_p=\frac{\omega _p}{2c}\chi _{ab}(\omega )\tilde{\Omega }_p, \end{aligned}$$

where $\tilde{x}\equiv \textrm{FT}(x)$. The solution is

11 $$\begin{aligned} \tilde{\Omega }_p=\tilde{\Omega }_{p0}\textrm{e}^{\textrm{i}[-\frac{\omega }{c}+\frac{\omega _p}{2c}\chi _{ab}(\omega )]z}, \end{aligned}$$

where $\tilde{\Omega }_{p0}\equiv \tilde{\Omega }_{p}(z=0,\omega )$ is the spectral shape of the pulse at the beginning of the sample. To obtain the shape of the pulse at the end of the medium, we inverse Fourier transform (IFT) the solution for $z=L$:

12 $$\begin{aligned} \Omega _p(L,t)=\textrm{IFT}\left( \tilde{\Omega }_{p0}\textrm{e}^{\textrm{i}[-\frac{\omega }{c}+\frac{\omega _p}{2c}\chi _{ab}(\omega )]L} \right) . \end{aligned}$$

The introduced equations can only be calculated analytically for simple cases, otherwise, numerical Fourier transformations are used. The approach is based on the probe susceptibility $\chi _{ab}$ evaluated in the regime linear with the probe field $\Omega _p$ [Eq. (3)], and limited to relatively weak probes to avoid the distortion of the pulse and population transfer. This is the frequency-domain part of the approach. The time-domain part involves the same procedure as the fully numerical solution, with the group index found based on Eq. (9). We compare the temporal shapes of the probe fields at the end of the sample in the absence or presence of the control field. To find the field at the end, instead of heavy simulations of the pulse propagation, we perform faster calculations in the frequency domain. The evaluated group index based on Eq. (9) corresponds to the group velocity of a spectrally broadened pulse with the carrier frequency detuning $\delta _p$ and approaches the analytical result, Eq. (8), in the limit of monochromatic fields.

In summary, the analytical solution given by Eq. (8) describes the group index of individual Fourier components of the probe pulse. The group index of a spectrally broad pulse can be calculated with Eq. (9) either in the fully numerical method or using the Fourier semi-analytical solution. The most general numerical method provides insight into the full population dynamics and thus can be time-consuming. It allows us to verify the validity of the $\sigma _{bb}=1$ assumption made for the other two methods. The Fourier approach, applied to the stationary case in the far-from-saturation regime, is a faster calculation method. In Supplement Sec. S3 the three methods are compared in detail.

## Results

In this section, we evaluate the optimal parameters of the control field for observing superluminality by introducing a two-dimensional figure of merit. The calculations are made for rubidium vapor parameters.

### Calculation parameters

The medium with length $L=4.91$ cm ($9.28\cdot 10^8$ a.u.) is filled with rubidium-87 vapor with states $\vert a \rangle = 5P_\frac{3}{2}$, $\vert b \rangle = 5S_\frac{1}{2}$ and $\vert c \rangle =5D_\frac{5}{2}$ and transition wavelengths $\lambda _{ba} = 780$ nm, $\lambda _{ac} = 776$ nm (The magnetic structure of the levels is neglected.). The lowest level is a ground state for the Rb atom with infinite lifetime while the middle level has the lifetime of $\tau _a=26$ ns ($1.08 \cdot 10^9$ a.u.) and the upper one of $\tau _c=238$ ns ($9.84 \cdot 10^9$ a.u.)^47^. The spontaneous emission rates are $\gamma _{aa}=2\pi /\tau _a=2\pi \cdot 6.1$ MHz ($9.26\cdot 10^{-10}$ a.u.) and $\gamma _{cc}=2\pi /\tau _c=2\pi \cdot 0.67$ MHz ($1.016\cdot 10^{-10}$ a.u.). The electric dipole moments for transitions $\vert b \rangle \rightarrow \vert a \rangle$ and $\vert a \rangle \rightarrow \vert c \rangle$ are $d_{ba}=5.1\cdot 10^{-29}$ C m (6 a.u.) and $d_{ac}=1.95\cdot 10^{-29}$ C m (2.3 a.u.)^48^. The concentration *N* is set to $10^{11}$ atoms/cm$^3$ ($1.5\cdot 10^{-14}$ a.u.).

### Selection of optimal parameters of CW control field

We analyse the probe output field as a function of control-field parameters to identify promising superluminal regimes. The probe propagates along the *z*-axis [minus signs in Eq. (2)] and the control field in the opposite direction [plus signs in Eq. (2)]. This is a common approach to reduce the Doppler broadening in experiments on $\Xi$ configuration systems^49^. The control parameters are the single-photon detuning $\delta _k$ and the amplitude $\mathcal {E}_k$ (or equivalently $\Omega _k$), affecting medium dispersion and absorption.

To optimise the control-field conditions, we define a figure of merit (FOM) that combines the requirements of the group index being below 1 and of a high transmission coefficient

13 $$\begin{aligned} \beta (\delta _k, \Omega _k; M)=\underbrace{\left( 1-n_g(\delta ^\text {min}, \delta _k, \Omega _k)\right) }_{\textrm{superluminality}}{\underbrace{\left( \textrm{e}^{-\frac{\omega _{p}}{2c}\chi _{ab}(\delta ^\text {min}, \delta _k, \Omega _k)L}\right) }_{\textrm{transmission}}}^M. \end{aligned}$$

For each set of control-field parameters $\delta _k$ and $\Omega _k$, we adjust the probe-field detuning to maximize $\beta$: $\delta ^\text {min}$ is the probe detuning that minimizes the $n_g(\delta , \delta _k, \Omega _k)$ in the vicinity of the two-photon resonance. The transmission coefficient is evaluated as the probe amplitude at the end of the sample relative to its input value. The FOM is constructed as a product of two quantities: The left parenthesis evaluates how much the group index differs from the vacuum value and needs to be positive for superluminality, while negative values of $\beta$ correspond to subluminal propagation. The higher the value of this parenthesis, the larger the group velocity of the pulse becomes. The right parenthesis describes transmission for the optimal detuning $\delta ^\text {min}$ of the probe field. The significance of transmission in the FOM is controlled by the parameter *M*. For $M=0$ transmission is not taken into account and the FOM only considers the degree of superluminality. For $M=1$, $\beta$ has maximal values for transmissions below 40% while the value of $M=4$ favours roughly 80% transmission, which better prevents pulse distortion.

**Figure 4:**
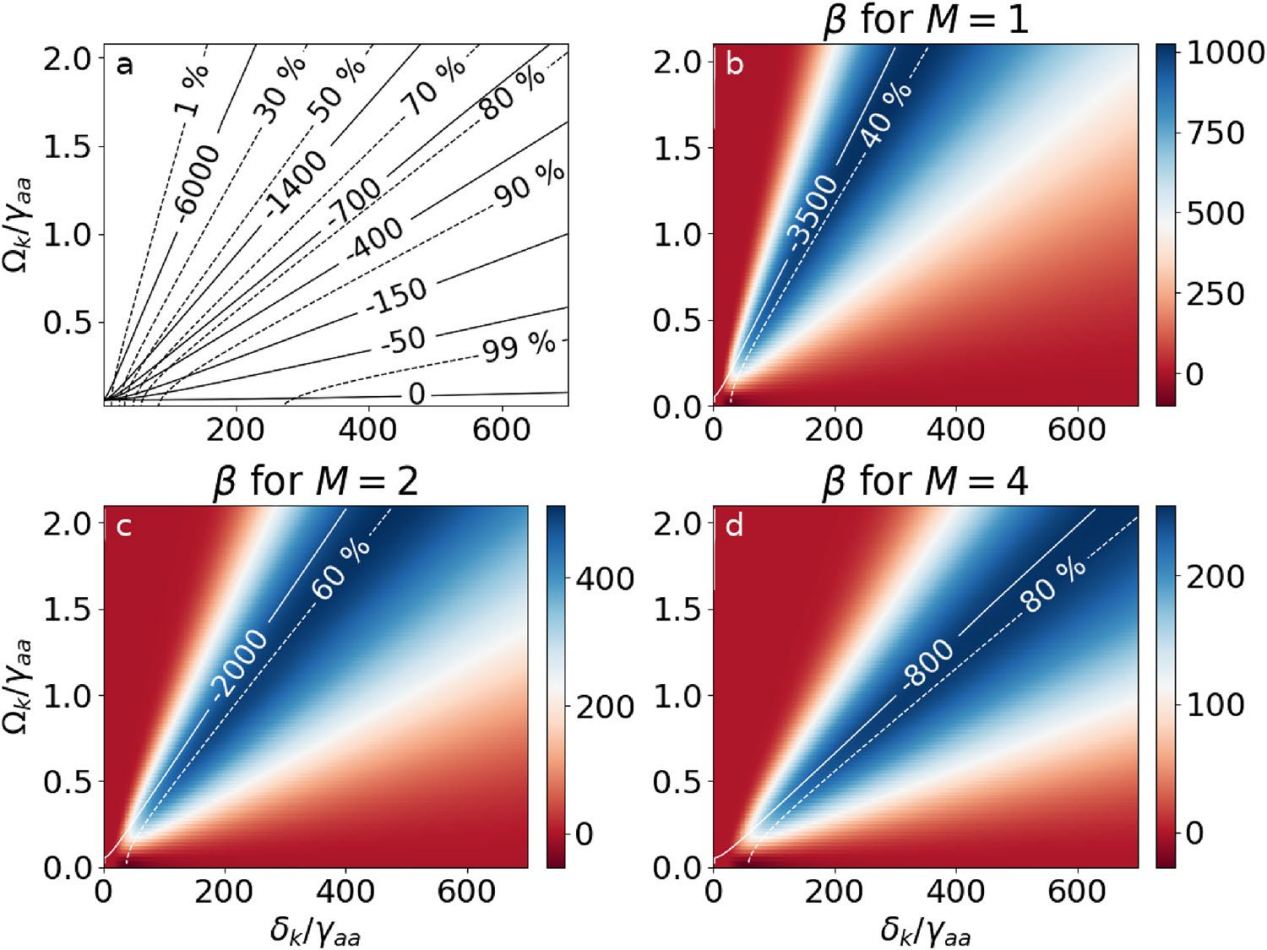
(**a**) Group index (solid lines) and transmission (dashed) as functions of the control-field Rabi frequency $\Omega _k$ and detuning $\delta _k$. (**b**–**d**) $\beta (\delta _k, \Omega _k; M)$ for different values of *M*. Group index and transmission near the FOM maximum are marked with white lines.

We now construct FOM maps as functions of the control-field parameters ($\delta _k, \Omega _k$) in Fig. 4. The single-photon detuning $\delta _k$ is hundreds of times larger than the width of the middle level ($\gamma _{aa}$) to suppress the influence of the single-photon resonance. Figure 4a shows the control-field detuning and amplitude plane with solid contour lines for the constant group index and dashed for transmission. Figure 4b–d presents $\beta$ for different values of *M*, with the colour scale indicating the FOM above or below half the maximal value shown in white. These plots guide the selection of control-field parameters for further investigation – the bluer the region, the better the conditions for the superluminal propagation. White lines mark the values of the group indices and transmission in the vicinity of the largest FOM values. For a higher value of *M* the FOM $\beta$ is maximized at larger transmission coefficients. Smaller values of *M* prioritize the minimized group index in the first bracket in Eq. (13). Thus, we find a trade-off between transmission and superluminal propagation velocity.

**Figure 5:**
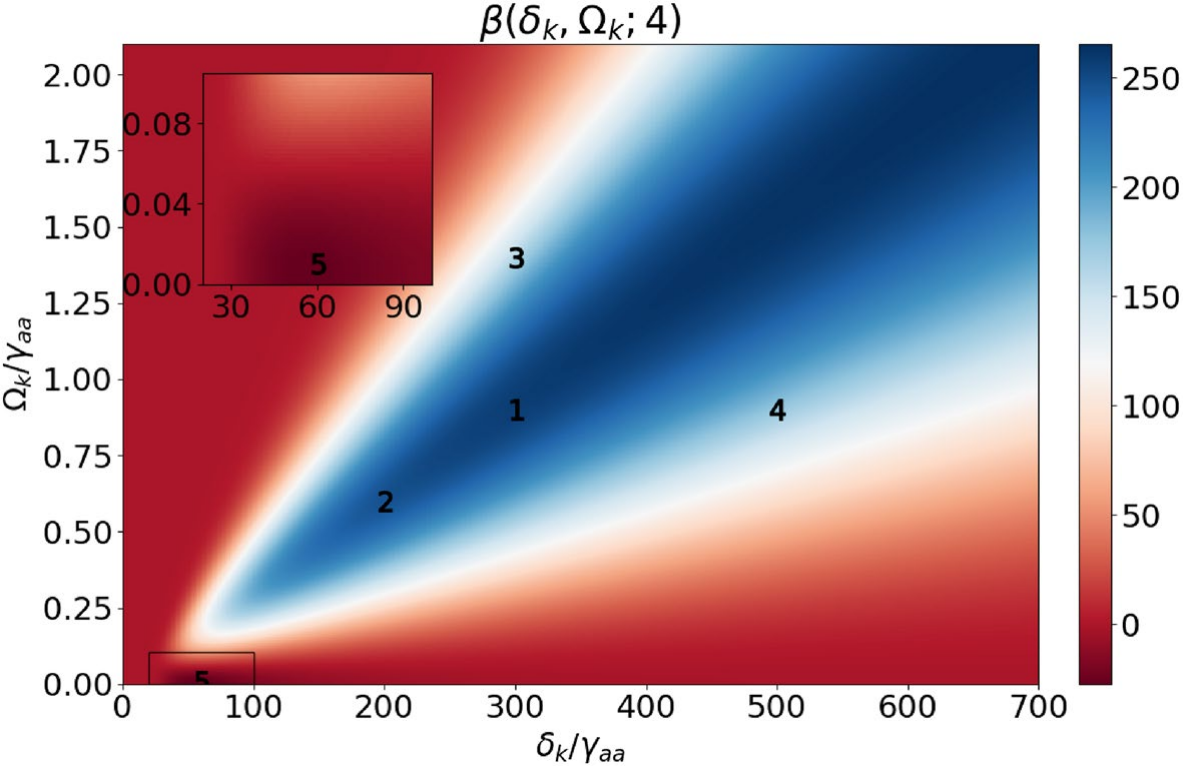
FOM $\beta$ for $M=4$ as a function of normalized detuning and Rabi frequency. Five points for further analysis are marked.

Five pairs of parameters are selected for further analysis with the FOM evaluated for $M=4$ (Fig. 5): **Point 1**, with the control-field Rabi frequency $\Omega _k=\gamma _{aa}$ and detuning $\delta _k=300\gamma _{aa}$ lies in the blue region, where small absorption and a high negative group index are observed. The conditions are favorable for superluminal propagation. FOM = 248.

**Point 2** demonstrates that for a weaker control-field amplitude $\Omega _k=0.5\gamma _{aa}$ a detuning $\delta _k=150\gamma _{aa}$ can be chosen to achieve a similar FOM = 238 as for **Point 1**.

For **Point 3** keeping the same detuning as in **Point 1**, we significantly increase the value of the control field $\Omega _k=1.5\gamma _{aa}$, which results in lower transmission but larger group index. FOM = 156.

For **Point 4** keeping the same amplitude of the control field as in **Point 1**, we significantly increase the detuning $\delta _k=500\gamma _{aa}$. This leads to greater transmission, but the value of the group index is closer to 1. FOM = 168.

**Point 5** belongs to the dark red region, where the parameter $\beta$ is negative (FOM = -27) in contrast to the previously analysed cases. In this case, the two-photon transition is suppressed by the single-photon transition. For the control-field Rabi frequency $\Omega _k=0.01\gamma _{aa}$ and $\delta _k=50\gamma _{aa}$, the resulting group index is a small correction at the background given by the single-photon resonance and the superluminal propagation conditions are not achieved.

We additionally introduce the fractional advancement parameter (*FA*), which is a percentage value of the pulse advancement $t_\text {adv} = t_g-t_0$ with respect to the pulse temporal width. It can be calculated directly from the Eq. (9) as

14 $$\begin{aligned} FA = 100\% \frac{t_\text {adv}}{2t_\text {HWHM}} = 100\%\frac{L}{c}\frac{n_g-1}{2t_\text {HWHM}}, \end{aligned}$$

where $t_\text {HWHM}$ is the temporal half-width-half-maximum of the pulse at the beginning of propagation.

For further calculations, we set the probe field $\Omega _p=0.05\gamma _{aa}$. We can consider it as a relatively weak field that fulfils requirements for the previously derived equations. The time step is set to $\Delta t = 1.28\cdot 10^{-4}\gamma _{aa}^{-1} = 3.34$ ps ($1.38\cdot 10^{5}$ a.u.) and the spatial step $\Delta x=c\Delta t=1.0$ mm ($1.89\cdot 10^{7}$ a.u.). This gives 50 spatial points along the sample.

### Broad and narrow pulse propagation analysis

**Figure 6:**
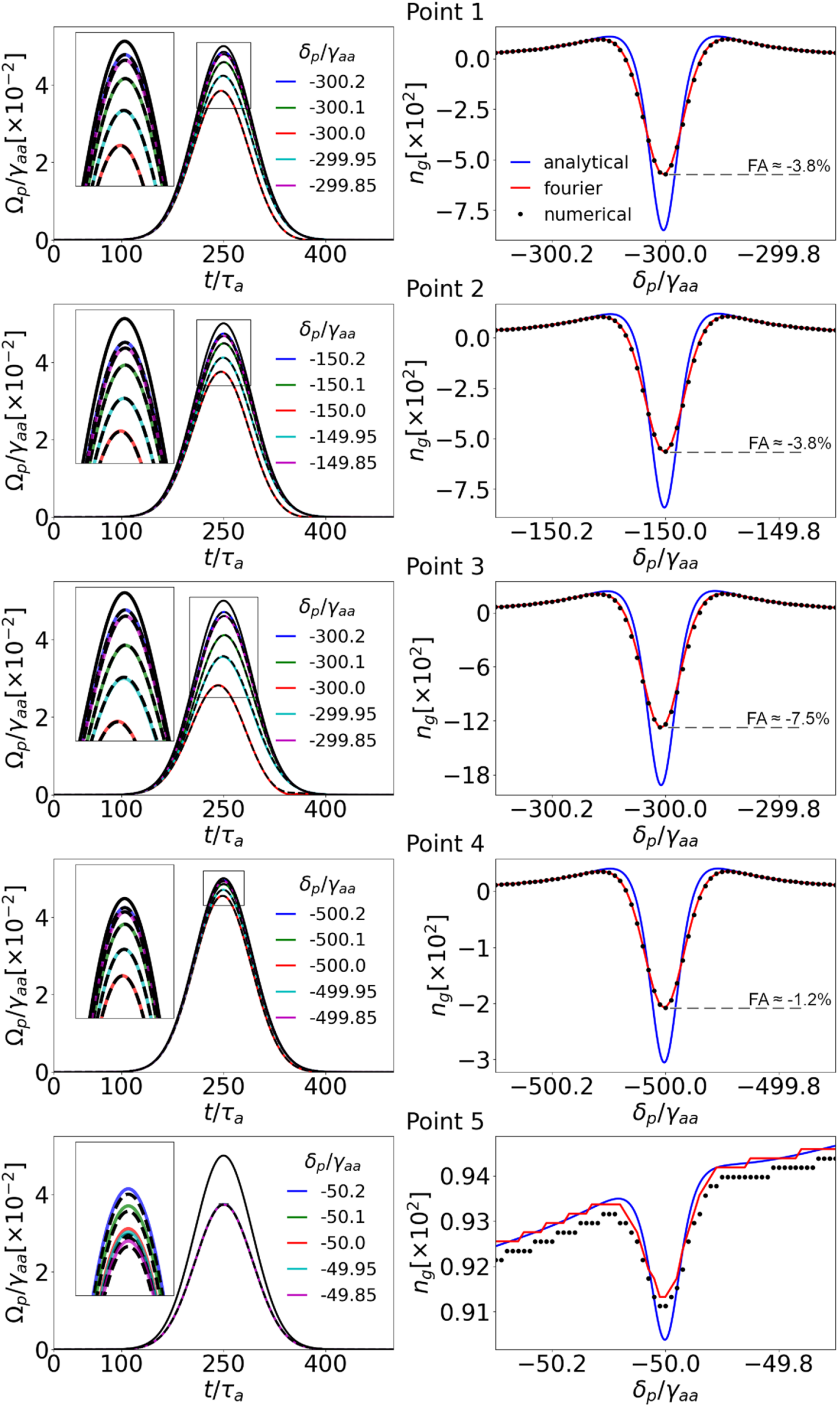
Transmitted-pulse temporal dependence (left column) and group-index spectral dependence versus the single-photon probe detuning (right column) at different two-photon resonance conditions for the long (spectrally narrow) pulse: **Point 1** $\Omega _k=\gamma _{aa}$, $\delta _k=300\gamma _{aa}$; **Point 2** $\Omega _k=0.5\gamma _{aa}$, $\delta _k=150\gamma _{aa}$; **Point 3** $\Omega _k=1.5\gamma _{aa}$, $\delta _k=300\gamma _{aa}$; **Point 4** $\Omega _k=\gamma _{aa}$, $\delta _k=500\gamma _{aa}$; **Point 5** $\Omega _k=0.01\gamma _{aa}$, $\delta _k=50\gamma _{aa}$. The colored solid lines shown in the left plots result from numerical calculations of pulses of different probe detunings $\delta _p$ near the two-photon transition. The dashed black lines are calculated with the Fourier approach for the same conditions. Solid black lines represent pulses travelling through free space of the same length and serve as reference. The right plots show resulting group indices for monochromatic waves (blue lines), Fourier (red lines), and numerical (dots) approaches. For the first four points, *FA* is also shown for the resonant pulse.

**Figure 7:**
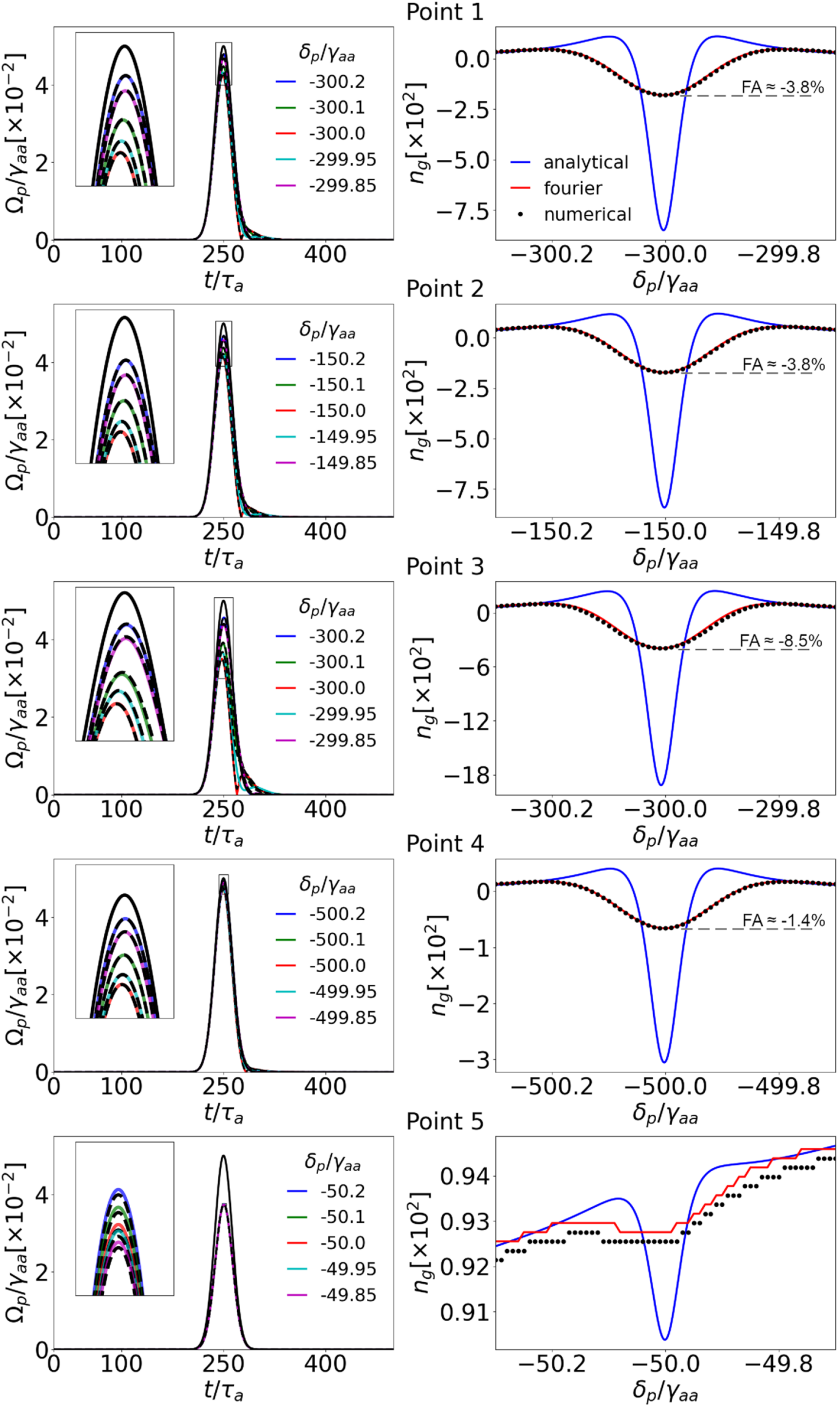
As Fig. 6 but for the short (spectrally broad) pulses.

We now investigate the propagation of the probe pulses under conditions defined by the five points above. Supplement Sec. S5.2 discusses monochromatic fields as a benchmark for other approaches. Such a wave has an infinitely narrow spectrum that provides the deepest dip in the group index curve, as demonstrated by the examples in Figs. 6 and 7 discussed below in detail. Here, we focus on Gaussian-shaped probe pulses as an important and experimentally feasible case. For a satisfactory resolution of numerical calculations described in Sec. 4.2 in the vicinity of the two-photon resonance we set $\Delta \omega _p=0.01\gamma _{aa}$. Then, calculations for discretized detunings $\delta _p=-\delta _k+m\cdot \Delta \omega _p$ are performed for $m\in \{-30, -29,..., 29, 30\}$, where $m=0$ corresponds to the two-photon resonance condition ($\delta _p + \delta _k = 0$). The other methods (see Secs. 4.1 and 4.3) are less time-consuming, so we could perform calculations with higher spectral resolution.

The control field modelled as a smoothed step pulse enters the sample first. After a stationary state is established, the probe pulse propagates in the opposite direction. The details of the control-field shape and parameters are discussed in Supplement Sec. S5.2. We performed calculations for Gaussian probe pulses with two different temporal lengths for all five $(\delta _k, \Omega _k)$ pairs, corresponding to the Points in Fig. 5. Such pulses propagate through the medium, and at the end, we collect their temporal shapes. The resulting peak amplitudes and positions are used to evaluate group indices [Eq. (9)], which are directly comparable with the analogous results for monochromatic waves [Eq. (8)].

#### Long Gaussian pulse

We first discuss Gaussian pulses of spectral width narrow with respect to the spontaneous-emission rate, characterized by the temporal $\text {HWHM}=50\tau _{a}=1.3$ $\mu$s ($5.4\cdot 10^{10}$ a.u.). The left-hand side of Fig. 6 presents the pulses at the end of the sample obtained numerically (solid lines) and calculated by the Fourier approach (dashed lines). All the results are in very good agreement, as the requirements of negligible population transfer and control-field modulation are met. Since we present the shapes for different values of the detuning, we capture pulses on the side of the absorption line (e.g. the blue ones) which are subliminal, as well as the superluminal ones near the resonance (e.g. red ones). Black solid lines show reference pulses travelling through free space.

On the right-hand side, the corresponding group indices are presented as functions of detunings $\delta _p/\gamma _{aa}$. Each point in black and along the red line corresponds to the group index of the Gaussian pulse with the carrier-frequency detuning $\delta _p$ and spectral width given above. On the contrary, each point along the blue curve, based on the analytical formula Eq. (8), describes a single-frequency component of the group index, which can be interpreted as the limiting case of pulses infinitely narrow spectrally. Results for the Gaussian pulses are slightly suppressed with respect to those of the monochromatic waves. The maximal values of *FA* obtained for resonant pulses are also marked on the right-hand side of Fig. 6.

For the most promising **Points 1** and **2**, the transmission on the two-photon resonance is around 80% as intended by setting the parameter $M=4$, while the group index drops to $n^\text {min}_g\approx -600$, which corresponds to the fractional advancement $FA \approx -3.8\%$. The similarity of these results can be explained by the fact that the absorption is tightly connected to the dispersion responsible for the group index. The two-photon resonance may be broadened due to the stronger control field; however, here this correction is marginal. For **Point 3**, as we go up in the FOM plot, the absorption is significantly greater, and the group index dip reaches $n^\text {min}_g\approx -1200$ ($FA\approx -7.5\%$); hence we observe the mentioned trade-off. The opposite situation is presented for **Point 4**: as we go to the right from **Point 1** in the plot, the absorption and group index are suppressed, resulting in a minimal value of $n^\text {min}_g\approx -200$ ($FA\approx -1.2\%$). The clearly subluminal propagation conditions provided by **Point 5**, intentionally chosen for demonstration purposes, lead to negligible absorption as the two-photon group index dip is a small correction on the left edge of a single-photon one. Additionally, we see discretization and discrepancy between numerical and analytical data. It indicates that our calculations at this point are at the numerical-accuracy limit: The arrival time difference as in Eq. (9) roughly corresponds to one time step $\Delta t$. All the calculations were performed for the same time and space steps ($\Delta t, \Delta z$), and so the last results serve as a limit reference in this particular setup. Naturally, decreasing these steps would increase the calculation time as well as the accuracy of the results.

In all cases, the results confirm conditions for superluminal propagation in three-level ladder systems with Rb vapors, except for one intentionally poor choice of parameters (Point 5). The results show negative group velocities and approach the limiting values of the group index achievable for monochromatic waves. These findings make Rb vapors a promising candidate medium for achieving superluminal propagation in experiments.

#### Short Gaussian pulse

To understand the effect of the pulse spectral width, we examine Gaussian pulses with $\text {HWHM} = 15\tau _{a}= 0.39$ $\mu$s ($1.6\cdot 10^{10}$ a.u.), roughly three times shorter than the previous one. Consequently, different spectral components are subject to slightly different medium response.

All the calculations were performed similarly as in the previous subsection. Figure 7 shows the resulting shapes at the end of the sample (left column) and the group indices dependence on the probe carrier frequency (right column). The absorption is much weaker as the spectral width is larger, and hence smaller part of the pulse efficiently interacts with the medium. For the same reason, shape deformations may appear, as can be seen on the right side of the pulse in the two-photon resonance (red curves). This is a limitation for the group-index evaluation as the more deformed the final pulse is, the less meaningful the group velocity becomes^46^. The wide spectral shape affects the propagation velocity as well: The group-index curve is broadened and suppressed compared to the monochromatic case. Nevertheless, the Fourier-based and numerical calculations are still in perfect agreement as no underlying assumptions are violated. It is also worth noticing that for Point 3, we obtain $n_g^\text {min}\approx -400$ which corresponds to $FA\approx -8.5\%$ at the transmission level well above 60%.

### Superluminal discussion

The examples presented in this section provide clear evidence for the superluminal propagation of light near the two-photon resonance. Based on the above analysis, one can formulate several important conclusions.

First, the analysis of Gaussian-pulse propagation shows that the calculation of the group index, performed in analogy to the refractive index, is justified as long as the spectral profile of the pulse is considered. As expected, results for narrow-spectrum pulses match those for monochromatic light. A larger discrepancy between the numerical and analytical results for both the absorption coefficient and the group index is observed for spectrally wider pulses. This may originate from the fact that even though the central frequency of the beam is far detuned from the single-photon transition, the spectral tail of such a pulse may still induce the transition, resulting in non-negligible absorption. This violates the assumption of the analytical treatment and calls for a description beyond the analytical level from Sec. 4.1.

Second, the group refractive index and accordingly the pulse velocity can be controlled by changing the amplitude of the control field $\Omega _k$ or its detuning $\delta _k$. By changing the control-field amplitude in exchange for a smaller transmission, we can significantly increase the group index and vice versa. Varying the control-field detuning allows one to switch between sub- and superluminal probe propagation.

Third, in the ladder scheme, the group index goes well below unity in the vicinity of the two-photon resonance. Simultaneously, different sets of parameters provide subluminal light propagation. Specifically, we can significantly reduce the pulse group velocity by simply detuning the probe field away from the two-photon resonance. The results are obtained in the case of probe light far detuned from the single-photon resonance, where the impact of the transition is small. Otherwise, for too small a separation between the dominant single-photon resonance and the two-photon resonance, the resulting group index is significantly increased. This can be seen in results for **Point 5**, with the group-index two-photon dip localized at the side slope of a single-photon one (Figs. 6 and 7).

Fourth, the results shown in Figs. 6 and 7 provide comparison between different approaches for Gaussian pulses. While pulse propagation can be simulated for different pulse shapes, group indices can be evaluated as long as there is a clearly distinguished point, which we can follow along the sample. For example, the rectangular shape is extremely difficult to investigate as the deformation on its edges would incorporate significant ambiguity in the position measurement. Hence, the propagation velocity would be indefinite.

Finally, with the medium in the ladder configuration, it is possible to use lower intensities of the probe field than in conventional two-level systems, where one operates in the linear absorption regime. This, given the concentration of the atoms in vapors and the sample size in a typical experiment (several centimetres), provides a promising scheme for experimental implementation. For this purpose, the introduced FOM map is a convenient tool for estimating propagation outcomes, especially for spectrally narrow pulses. As a quantity simple and fast to calculate, the FOM can be used to identify potentially interesting parameter regimes before performing time-consuming simulations.

## Conclusions

The theoretical investigations presented in this work aimed at analysing superluminal light propagation in three-level systems under the two-photon absorption conditions. The analysis was performed with three different approaches, both analytical and numerical, concerning both the efficiency and correctness of the results. The results allowed us to formulate and verify the applicability conditions of the semi-analytical approach based on the spectral decomposition of the pulses. Based on these three approaches, the group index for different sets of the probe- and control-light parameters was calculated in a realistic scheme of rubidium vapor. To quantitatively compare the results for different control-field parameters, the figure-of-merit $\beta (\delta _k, \Omega _k; M)$ was introduced. It accounted for both the dispersion and absorption of the medium and allowed us to identify the conditions under which the optimal propagation was achieved.

The versatility of the numerical and the Fourier methods allows for future analysis of more complex scenarios. One can envision light propagation with a time-dependent control field, for which the properties of the medium change, e.g., switching between sub- and superluminal regimes for the probe pulses. Pulse reshaping effects could be discussed in the regimes in which the group velocity is no longer a valid quantity. Another interesting case is the nonlinear regime when the medium supports the refractive index dependence on the probe intensity in the Kerr effect, related to the formation of optical solitons, optical vortices or applications of optical switches and logic gates for all-optical computing.

### Supplementary Information

Supplementary Information.

## Data Availability

The data underlying the results presented in this paper are publicly available at 10.18150/Z5U8MG
