# Supplementary material for: Superluminal light propagation in a three-level ladder system
Source: Sci Rep. 2024 Jul 2;14:15151. doi: 10.1038/s41598-024-62220-x (PMC11220075; doi:10.1038/s41598-024-62220-x)
Supplement: Supplementary file 1 — Supplementary Information. [file 41598_2024_62220_MOESM1_ESM.pdf]

Supplement: Superluminal light propagation in a three-level ladder system

Piotr Gładysz[1,*], Szymon Pustelny[2], and Karolina Słowik[1]

[1]Institute of Physics, Faculty of Physics, Astronomy and Informatics, Nicolaus Copernicus University in Toruń, Grudziadzka 5, 87-100 Toruń, Poland
[2]Institute of Physics, Jagiellonian University, Łojasiewicza 11, 30-348 Kraków, Poland
*glad@doktorant.umk.pl

Here, we discuss in detail the following topics:
S1. Derivation of the Bloch-Maxwell equations.
We derive general equations for the evolution of the one-dimensional three-level media and for the propagation of the electric fields.
S2. Spontaneous emission and dephasing.
We evaluate the matrix forms of the operators corresponding to the spontaneous emission and dephasing processes in all three types of three-level systems considered in the main text.
S3. Numerical methods.
We discuss numerical methods used for simulations and briefly describe the parallelization of the calculations.
S4. Derivation of electric susceptibilities for three-level systems.
We provide a comparison of three-level configurations in terms of electric susceptibilities in each case.
S5. Validation of the approaches.
We present evidence that the approaches introduced in the main text are valid and give the same results under various conditions.

S1 Derivation of the Bloch-Maxwell equations

In our analysis, we focus on a 1D atomic medium, with atoms modelled as three-level systems. The medium is described with the formalism based on spatiotemporal density matrix $\rho(z,t)$, whose evolution is given by the Lindblad master equation that includes relaxation processes (spontaneous emission or dephasing)

$$\mathrm{i}\hbar\dot{\rho}(z,t) = [H(z,t),\rho(z,t)] + \mathscr{L}(\rho(z,t)), \tag{S1}$$

where $\hbar$ is the reduced Planck constant and $\mathscr{L}$ is the Lindblad operator. In the energy basis $\{|c\rangle, |a\rangle, |b\rangle\}$, the matrix representation of the Hamiltonian H is given by

$$H = \begin{bmatrix} \hbar\omega_c & -\vec{E}_k(z,t)\vec{d}_{ca} & 0 \\ -\vec{E}_k(z,t)\vec{d}_{ac} & \hbar\omega_a & -\vec{E}_p(z,t)\vec{d}_{ab} \\ 0 & -\vec{E}_p(z,t)\vec{d}_{ba} & \hbar\omega_b \end{bmatrix}. \tag{S2}$$

The above form of the Hamiltonian reveals that only electric-dipole interactions are considered in our treatment (the electric-dipole approximation). The electric-dipole moment elements $\vec{d}_{ij}$ are responsible for the transitions between the states $|i\rangle$ and $|j\rangle$, where $i,j \in \{a,b,c\}$ and $i \neq j$. In all cases, the transition between levels $|c\rangle$ and $|b\rangle$ is forbidden, $\vec{d}_{bc} = \vec{d}_{cb} = \vec{0}$. Additionally, for the V type medium, we set $\hbar\omega_a = 0$ while for the two other cases, $\hbar\omega_b = 0$ and the energies of the other levels are evaluated with respect to the corresponding ground state.

The general expression for the Lindblad operator is

$$\mathscr{L}(\rho) = 2\mathrm{i}\hbar\sum_{ij}\gamma'_{ij}\left[L_{ij}\rho L^\dagger_{ij} - \frac{1}{2}\left(L^\dagger_{ij}L_{ij}\rho + \rho L^\dagger_{ij}L_{ij}\right)\right], \tag{S3}$$

where $i,j \in \{a,b,c\}$ such that $\omega_j > \omega_i$ and $L_{ij} = |i\rangle\langle j|$ are flip operators, describing spontaneous emission, while γ'_{ij} are the corresponding relaxation rates. Dephasing can be included by setting $i = j$[1] (for more information see Sec. S2.4). For each type three-level system, there are only two non-zero rates describing spontaneous emission: γ'_{ab} and γ'_{ac}, γ'_{ba} and γ'_{ca}, γ'_{ba} and

γ'_{ac} for V, Λ, and Ξ, respectively. Since for all the cases the matrix forms of $\mathscr{L}(\rho)$ are significantly different, we derive their explicit forms in Sec. S2.

The levels $|a\rangle$ and $|b\rangle$ are coupled via the electric field of the probe light given by

$$\vec{E}_p(z,t) = \mathscr{E}_p(z,t)\vec{e}_p\cos(k_pz \pm \omega_pt). \tag{S4}$$

Similarly, the control field

$$\vec{E}_k(z,t) = \mathscr{E}_k(z,t)\vec{e}_k\cos(k_kz \pm \omega_kt) \tag{S5}$$

couples the $|a\rangle$ and $|c\rangle$ levels. Here $\mathscr{E}_p$ and $\mathscr{E}_k$ are the envelopes of the fields, $\vec{e}_p$ and $\vec{e}_k$ are the corresponding polarization vectors, ω_p and ω_k stand for the carrier frequencies, and k_p and k_k are the projections of the wavevectors onto the z-axis. Additionally, since we keep calculations general, we allow the fields to propagate in either of the two directions along the z-axis, which is represented by the $\pm$ signs in the argument of cosine functions.

To derive equations for each density matrix elements ρ_{ij}, we introduce the substitutions

$$\rho_{aa}(z,t) = \sigma_{aa}(z,t), \tag{S6a}$$
$$\rho_{bb}(z,t) = \sigma_{bb}(z,t), \tag{S6b}$$
$$\rho_{ba}(z,t) = \sigma_{ba}(z,t)\mathrm{e}^{\mp\alpha_p\mathrm{i}(k_pz\pm\omega_pt)}, \tag{S6c}$$
$$\rho_{ac}(z,t) = \sigma_{ac}(z,t)\mathrm{e}^{\pm\alpha_k\mathrm{i}(k_kz\pm\omega_kt)}, \tag{S6d}$$
$$\rho_{bc}(z,t) = \sigma_{bc}(z,t)\mathrm{e}^{\mp\alpha_p\mathrm{i}(k_pz\pm\omega_pt)}\mathrm{e}^{\pm\alpha_k\mathrm{i}(k_kz\pm\omega_kt)}, \tag{S6e}$$

where the lower or upper sets of signs should be used according to the propagation-direction choice from Eqs. (S4) and (S5). To distinguish different three-level systems, we introduced the parameters α_p and α_k that define signs for different cases:

- for V system: $\alpha_p = \alpha_k = +1$,
- for Λ system: $\alpha_p = \alpha_k = -1$,
- for Ξ system: $\alpha_p = -1,\ \alpha_k = +1$.

The substitution allows us to perform the rotating-wave approximation (RWA) and simplify the evolution [Eq. (S1)] by removing the rapidly oscillating terms. The resulting set of the Bloch equations is

$$\dot{\sigma}_{aa} = \mathrm{i}\Omega_p\sigma_{ba} - \mathrm{i}\Omega_p\sigma_{ab} - \mathrm{i}\Omega_k\sigma_{ac} + \mathrm{i}\Omega_k\sigma_{ca} + \mathscr{L}_{aa}, \tag{S7a}$$
$$\dot{\sigma}_{cc} = \mathrm{i}\Omega_k\sigma_{ac} - \mathrm{i}\Omega_k\sigma_{ca} + \mathscr{L}_{cc}, \tag{S7b}$$
$$\dot{\sigma}_{ba} = \alpha_p\mathrm{i}\delta_p\sigma_{ba} - \mathrm{i}\Omega_p(1-\sigma_{cc}-2\sigma_{aa}) - \mathrm{i}\Omega_k\sigma_{bc} + \mathscr{L}_{ba}, \tag{S7c}$$
$$\dot{\sigma}_{ac} = -\alpha_k\mathrm{i}\delta_k\sigma_{ac} + \mathrm{i}\Omega_p\sigma_{bc} - \mathrm{i}\Omega_k(\sigma_{aa}-\sigma_{cc}) + \mathscr{L}_{ac}, \tag{S7d}$$
$$\dot{\sigma}_{bc} = (\alpha_p\mathrm{i}\delta_p - \alpha_k\mathrm{i}\delta_k)\sigma_{bc} + \mathrm{i}\Omega_p\sigma_{ac} - \mathrm{i}\Omega_k\sigma_{ba} + \mathscr{L}_{bc}, \tag{S7e}$$

where we introduced the Rabi frequencies $\Omega_p = \mathscr{E}_p\vec{e}_p\cdot\vec{d}_{ba}/2\hbar$ and $\Omega_k = \mathscr{E}_k\vec{e}_k\cdot\vec{d}_{ac}/2\hbar$ of the probe and control fields, respectively. We also assumed real values of the electric dipole moments, $\vec{d}_{ij} = \vec{d}_{ji}$ and preservation of atomic population ($\sum_i\sigma_{ii} = 1$). The detunings from single-photon resonances are defined as $\delta_p = \omega_p - \omega_{ba}$ for the probe field and $\delta_k = \omega_k - \omega_{ac}$ for the control field.

The propagation of the fields along the medium can be fully described by the wave equation

$$\left(-\partial_z^2 + \frac{1}{c^2}\partial_t^2\right)\left[\vec{E}_p(z,t) + \vec{E}_k(z,t)\right] = -\mu_0\partial_t^2\vec{P}(z,t). \tag{S8}$$

Here, $\vec{P}$ stands for the polarization induced in the medium by the fields and μ_0 is the vacuum permeability. The relation between this macroscopic quantity and the quantum state of the medium is given by $\vec{P}(z,t) = N\,\mathrm{Tr}\left(\rho\vec{d}\right)$, where N is the concentration of atoms. We can now apply RWA and the slowly-varying-envelope approximation (SVEA) to Eq. (S8). The latter is used if one can assume that the field envelopes $\mathscr{E}_{p,k}$ change slowly in time (space) compared to the rapid oscillations at the carrier

frequency. In such a case, time derivatives of σ_{ij} can be neglected, as $|\dot{\sigma}_{ij}| \ll |\omega\sigma_{ij}|$, and the wave equation can be separated into a set of first-order equations for the probe and control fields[2]

$$(\partial_t \pm c\partial_z)\,\Omega_p = \pm\alpha_p \mathrm{i}\frac{N\omega_p|d_{ba}|^2}{2\hbar\varepsilon_0}\sigma_{ba}, \tag{S9a}$$

$$(\partial_t \pm c\partial_z)\,\Omega_k = \pm\alpha_k \mathrm{i}\frac{N\omega_k|d_{ac}|^2}{2\hbar\varepsilon_0}\sigma_{ac}, \tag{S9b}$$

where ε_0 is the vacuum permittivity. As a result, probe- and control-field dynamics depend on the coherences in the medium originating from the interaction with the fields. Equations (S7) and (S9) are known in the literature as Bloch-Maxwell equations.

S2 Spontaneous emission and dephasing

In the considered case, the Bloch equations, given in Eqs. (S7), contain terms with $\mathscr{L}_{ij}$. The terms are derived from the master equation Eq. (S3) and are responsible for spontaneous emission. Here, we derive the exact forms of these terms for all the energy-level schemes considered in the manuscript.

S2.1 V-type

The spontaneous emission is described by the two flip operators $L_{ab} = |a\rangle\langle b|$ and $L_{ac} = |a\rangle\langle c|$ (the transition between the levels $|c\rangle$ and $|b\rangle$ is forbidden). Hence, the Lindblad operator takes the form[3]

$$\begin{aligned}\mathscr{L}^V(\rho) = 2\mathrm{i}\hbar\Big\{ &\gamma'_{ab}\left[L_{ab}\rho L_{ab}^\dagger - \frac{1}{2}\left(L_{ab}^\dagger L_{ab}\rho + \rho L_{ab}^\dagger L_{ab}\right)\right] \\ &+\gamma'_{ac}\left[L_{ac}\rho L_{ac}^\dagger - \frac{1}{2}\left(L_{ac}^\dagger L_{ac}\rho + \rho L_{ac}^\dagger L_{ac}\right)\right]\Big\}.\end{aligned} \tag{S10}$$

The matrix form of the operator in the eigenbasis $\{|c\rangle, |a\rangle, |b\rangle\}$ is

$$\mathscr{L}^V \doteq \mathrm{i}\hbar\begin{bmatrix} -2\gamma'_{ac}\rho_{cc} & -\gamma'_{ac}\rho_{ca} & -(\gamma'_{ab}+\gamma'_{ac})\rho_{cb} \\ -\gamma'_{ac}\rho_{ac} & 2\gamma'_{ab}\rho_{bb}+2\gamma'_{ac}\rho_{cc} & -\gamma'_{ab}\rho_{ab} \\ -(\gamma'_{ab}+\gamma'_{ac})\rho_{bc} & -\gamma'_{ab}\rho_{ba} & -2\gamma'_{ab}\rho_{bb}\end{bmatrix}. \tag{S11}$$

Usually, this matrix is presented in a different form with use of the rewritten variables as below

$$\mathscr{L}^V \doteq \mathrm{i}\hbar\begin{bmatrix} -\gamma_{cc}\rho_{cc} & -\gamma_{ac}\rho_{ca} & -\gamma_{bc}\rho_{cb} \\ -\gamma_{ac}\rho_{ac} & \gamma_{bb}\rho_{bb}+\gamma_{cc}\rho_{cc} & -\gamma_{ab}\rho_{ab} \\ -\gamma_{bc}\rho_{bc} & -\gamma_{ab}\rho_{ba} & -\gamma_{bb}\rho_{bb}\end{bmatrix}, \tag{S12}$$

where $\gamma_{cc} = 2\gamma'_{ac}$, $\gamma_{bb} = 2\gamma'_{ab}$, $\gamma_{ab} = \gamma'_{ab} = \frac{1}{2}\gamma_{bb}$, $\gamma_{ac} = \gamma'_{ac} = \frac{1}{2}\gamma_{cc}$, and $\gamma_{bc} = \gamma'_{ab} + \gamma'_{ac} = \frac{1}{2}(\gamma_{bb}+\gamma_{cc})$. Here, γ_{ii} is the relaxation rate of the state $|i\rangle$, while γ_{ij}, $i \neq j$, are the terms responsible for the decay of coherences, i.e., the off-diagonal elements of the density matrix. If spontaneous emission is the only source of decoherence, we can express the decoherence rates by the spontaneous emission rates:

$$\mathscr{L}^V \doteq \mathrm{i}\hbar\begin{bmatrix} -\gamma_{cc}\rho_{cc} & -\frac{1}{2}\gamma_{cc}\rho_{ca} & -\frac{1}{2}(\gamma_{bb}+\gamma_{cc})\rho_{cb} \\ -\frac{1}{2}\gamma_{cc}\rho_{ac} & \gamma_{bb}\rho_{bb}+\gamma_{cc}\rho_{cc} & -\frac{1}{2}\gamma_{bb}\rho_{ab} \\ -\frac{1}{2}(\gamma_{bb}+\gamma_{cc})\rho_{bc} & -\frac{1}{2}\gamma_{bb}\rho_{ba} & -\gamma_{bb}\rho_{bb}\end{bmatrix}. \tag{S13}$$

S2.2 Λ-type

For the Λ energy configuration, the following flip operators are important for the spontaneous emission: $L_{ba} = |b\rangle\langle a|$ and $L_{ca} = |c\rangle\langle a|$. Thus, we have

$$\begin{aligned}\mathscr{L}^\Lambda(\rho) = 2\mathrm{i}\hbar\Big\{ &\gamma'_{ab}\left[L_{ba}\rho L_{ba}^\dagger - \frac{1}{2}\left(L_{ba}^\dagger L_{ba}\rho + \rho L_{ba}^\dagger L_{ba}\right)\right] \\ &+\gamma'_{ac}\left[L_{ca}\rho L_{ca}^\dagger - \frac{1}{2}\left(L_{ca}^\dagger L_{ca}\rho + \rho L_{ca}^\dagger L_{ca}\right)\right]\Big\},\end{aligned} \tag{S14}$$

which in the matrix form is given by

$$\mathscr{L}^{\Lambda} \doteq \mathrm{i}\hbar \begin{bmatrix} 2\gamma'_{ac}\rho_{aa} & -(\gamma'_{ab}+\gamma'_{ac})\rho_{ca} & 0 \\ -(\gamma'_{ab}+\gamma'_{ac})\rho_{ac} & -2(\gamma'_{ab}+\gamma'_{ac})\rho_{aa} & -(\gamma'_{ab}+\gamma'_{ac})\rho_{ab} \\ 0 & -(\gamma'_{ab}+\gamma'_{ac})\rho_{ba} & 2\gamma'_{ab}\rho_{aa} \end{bmatrix}. \tag{S15}$$

Here, we can rewrite this matrix in analogy to the V scheme, but an additional assumption is needed. Usually, the lower levels are energetically close to each other in comparison with the upper level, and we assume $\gamma'_{ab} \approx \gamma'_{ac}$. Under these assumptions, we have new substitutions $\gamma_{aa} = 2(\gamma'_{ab}+\gamma'_{ac})$, $\gamma_{ab} = \gamma_{ac} = \gamma'_{ab}+\gamma'_{ac} = \frac{1}{2}\gamma_{aa}$, and $\gamma_{bc} = 0$. These lead to the final matrix form of the Λ-scheme Lindblad operator

$$\mathscr{L}^{\Lambda} \doteq \mathrm{i}\hbar \begin{bmatrix} \frac{1}{2}\gamma_{aa}\rho_{aa} & -\frac{1}{2}\gamma_{aa}\rho_{ca} & 0 \\ -\frac{1}{2}\gamma_{aa}\rho_{ac} & -\gamma_{aa}\rho_{aa} & -\frac{1}{2}\gamma_{aa}\rho_{ab} \\ 0 & -\frac{1}{2}\gamma_{aa}\rho_{ba} & \frac{1}{2}\gamma_{aa}\rho_{aa} \end{bmatrix}. \tag{S16}$$

S2.3 Ξ-type

As previously, only two flip operators ($L_{ba} = |b\rangle\langle a|$ and $L_{ac} = |a\rangle\langle c|$) describe the spontaneous emission in the Ξ system:

$$\begin{aligned} \mathscr{L}^{\Xi}(\rho) = 2\mathrm{i}\hbar \Big\{ & \gamma'_{ab}\left[L_{ba}\rho L_{ba}^{\dagger} - \frac{1}{2}\left(L_{ba}^{\dagger}L_{ba}\rho + \rho L_{ba}^{\dagger}L_{ba}\right)\right] \\ & \gamma'_{ac}\left[L_{ac}\rho L_{ac}^{\dagger} - \frac{1}{2}\left(L_{ac}^{\dagger}L_{ac}\rho + \rho L_{ac}^{\dagger}L_{ac}\right)\right]\Big\} \end{aligned} \tag{S17}$$

and the matrix form is

$$\mathscr{L}^{\Xi} \doteq \mathrm{i}\hbar \begin{bmatrix} -2\gamma'_{ac}\rho_{cc} & -(\gamma'_{ab}+\gamma'_{ac})\rho_{ca} & -\gamma'_{ac}\rho_{cb} \\ -(\gamma'_{ab}+\gamma'_{ac})\rho_{ac} & -2\gamma'_{ab}\rho_{aa}+2\gamma'_{ac}\rho_{cc} & -\gamma'_{ab}\rho_{ab} \\ -\gamma'_{ac}\rho_{bc} & -\gamma'_{ab}\rho_{ba} & 2\gamma'_{ab}\rho_{aa} \end{bmatrix}. \tag{S18}$$

By substituting $\gamma_{aa} = 2\gamma'_{ab}$, $\gamma_{cc} = 2\gamma'_{ac}$, $\gamma_{ab} = \gamma'_{ab} = \frac{1}{2}\gamma_{aa}$, $\gamma_{ac} = \gamma'_{ab}+\gamma'_{ac} = \frac{1}{2}(\gamma_{aa}+\gamma_{cc})$, and $\gamma_{bc} = \gamma'_{ac} = \frac{1}{2}\gamma_{cc}$, we obtain

$$\mathscr{L}^{\Xi} \doteq \mathrm{i}\hbar \begin{bmatrix} -\gamma_{cc}\rho_{cc} & -\frac{1}{2}(\gamma_{aa}+\gamma_{cc})\rho_{ca} & -\frac{1}{2}\gamma_{cc}\rho_{cb} \\ -\frac{1}{2}(\gamma_{aa}+\gamma_{cc})\rho_{ac} & -\gamma_{aa}\rho_{aa}+\gamma_{cc}\rho_{cc} & -\frac{1}{2}\gamma_{aa}\rho_{ab} \\ -\frac{1}{2}\gamma_{cc}\rho_{bc} & -\frac{1}{2}\gamma_{aa}\rho_{ba} & \gamma_{aa}\rho_{aa} \end{bmatrix}. \tag{S19}$$

S2.4 Extended description

To include the additional dephasing processes, we can modify the matrices (S13), (S16), and (S19) by adding the dephasing matrix

$$\mathscr{L}^{\mathrm{dep}} \doteq \mathrm{i}\hbar \begin{bmatrix} 0 & -\gamma_{ac}^{\mathrm{dep}}\rho_{ca} & -\gamma_{bc}^{\mathrm{dep}}\rho_{cb} \\ -\gamma_{ac}^{\mathrm{dep}}\rho_{ac} & 0 & -\gamma_{ab}^{\mathrm{dep}}\rho_{ab} \\ -\gamma_{bc}^{\mathrm{dep}}\rho_{bc} & -\gamma_{ab}^{\mathrm{dep}}\rho_{ba} & 0 \end{bmatrix}, \tag{S20}$$

where $\gamma_{ij}^{\mathrm{dep}}$ stands for the dephasing rates obtained from the master equation for the given dephasing operator, e.g., $L^{\mathrm{dep}} = \frac{1}{\sqrt{2}}(|i\rangle\langle i| - |j\rangle\langle j|)$ where i and j correspond to the involved states.

Despite the similar form of the Lindblad equations in different systems, the dynamics they represent turns out different. In the Λ-type model, spontaneous emission does not disrupt the coherences between the lower levels, and hence the coherent effects are the strongest. In the Ξ- and V-type systems, spontaneous emission influences the coherences, and hence the coherent effects are weaker. Based on Eqs. (S13, S16, and S19), in the V-type the suppression of coherence between the $|b\rangle$ and $|c\rangle$ states is the fastest among the three configurations due to the dependence on the spontaneous emission rates from both upper levels, while in the Ξ-type only the highest level contributes. These observations are reflected in the susceptibility profiles shown in the main text in Fig. 2 in Sec. 3.

S3 Numerical methods

For the propagation problem presented in the paper, we solve the Bloch-Maxwell equations [Eqs. (1) and (2) in the main text]. This can be done analytically for a few simple cases, but in general, the numerical approach is required. The calculations of the dynamics can be made in the discrete-time domain by solving the equations for the whole medium in successive time steps.

The equations describing the evolution of the medium [Eqs. (1)] are position-dependent. In the considered case, the medium consists of non-interacting three-level systems, and hence the dynamics of each of them can be calculated separately with the use of the Runge-Kutta fourth-order method[4]. This approach provides an accuracy proportional to the fourth order of the time step $(\Delta t)^4$.

For the calculations of the evolution of the probe and control pulses, we use the Lax-Wendroff method[5]. It is based on finite differences and has an accuracy proportional to the squared time step $(\Delta t)^2$. The general approach is based on solving the equation

$$\partial_t f(z,t) + A\partial_z f(z,t) = s(z,t), \tag{S21}$$

where A is a constant, f is a solution we are looking for, and s is a source term. For the solution, we introduce the discretization $t \to t_p = t_0 + p\Delta t$ for $p = 0,1,2,...$ and $z \to z_q = z_0 + q\Delta z$ for $q = 0,1,2,...,n$. Additionally, we choose $\Delta z = A\Delta t$. To simplify, we write $f(z,t) = f(t,z) \to f(t_p,z_q) \equiv f_{p,q}$. To calculate $f_{p+1,q}$ for all q, we use the formula[6]

$$f_{p+1,q} = f_{p,q} \pm \frac{1}{2}(f_{p,q+1} - f_{p,q-1}) + \frac{1}{2}(f_{p,q+1} - 2f_{p,q} + f_{p,q-1}) + \Delta t s_{p,q}, \tag{S22}$$

where the $\pm$ sign has to be set to minus for propagation along the z-axis and to plus otherwise. Since it is a first-order differential equation, we have to know the initial conditions $f_{0,q}$ for all q and the boundary conditions $f_{p,0}$, $f_{p,n}$ for all p.

Comparing Eq. (S21) with Eq. (2), we can adapt the described method to the considered case. Specifically, the function s can be identified as a solution of the Bloch equations, precisely the coherences σ_{ij} from Eq. (2) driving the probe and control field.

We also implemented multiprocessing in the code so that we can calculate real-life samples in minutes instead of hours or days. Instead of calculating everything in a loop, we divide the medium into smaller pieces and do the calculations separately, exchanging only the values from the overlapping points on the edges between them. To calculate a point $f_{p+1,q}$, according to Eq. (S22), we have to know three points ($f_{p,q-1}$, $f_{p,q}$, and $f_{p,q+1}$) from the previous time step (see Fig. S1).

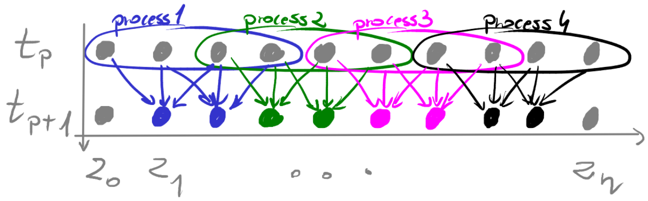

Figure S1. Representative ten space points and four processes (different colors). Each point in time step t_{p+1} requires three points from the previous step t_p (presented by arrows). Because of that, if we divide the medium into four parts, to perform proper calculations each part has to contain two more points from t_p than the resulting field has in t_{p+1}. The boundary conditions provide values for the edge points for all times t_p, so that the same number of points in space is used for each time step. This process repeats for all time steps used in the calculations.

S4 Derivation of electric susceptibilities for three-level systems

In the frequency domain, Eqs. (2) can be rewritten in terms of the electric susceptibility of the medium[2]. The probe-field susceptibility χ_p can be evaluated by a direct comparison of medium linear polarization components at the probe-field frequency $P(\delta) = \varepsilon_0\chi(\delta)\mathscr{E}_p(\delta)$ and the microscopic description $P(\delta) = N\,\mathrm{Tr}[\sigma_{ab}(\delta)d_{ba}]$ for the Λ and Ξ systems and $P(\delta) = N\,\mathrm{Tr}[\sigma_{ba}(\delta)d_{ab}]$ for the V system. For convince, here the quantities are given using detuning $\delta = \omega - \omega_{ab}$ and ωs are spectral components of the pulse.[1] The vector notation is omitted since we assume that all vectors are co-linear. In our analysis, $\sigma_{ab(ba)}$ is assumed to give rise only to the relevant component of the polarization at the probe frequency, i.e., it does not couple other transitions.

The spectral components of the density-matrix elements $\sigma_{ij}(\delta)$ correspond to the stationary solutions obtained by replacing the derivatives in Eqs. (1) by zeros. The resulting expression for electric susceptibility for the probe field reads as

$$\chi_p(\delta,\delta_k,\Omega_p,\Omega_k) = \frac{N|d_{ab}|^2}{\hbar\varepsilon_0}\frac{\sigma_p^{\mathrm{stat}}(\delta,\delta_k,\Omega_k,\Omega_p)}{\Omega_p}, \tag{S23}$$

[1] The Fourier variable ω which we work with in the frequency domain may be confused with the carrier frequency of the probe pulse ω_p (similarly, the detuning δ can be confused with the detuning from the central frequency δ_p). This is only valid for monochromatic beams; when considering a pulse with many spectral components, ωs are distributed around the carrier frequency ω_p. We emphasize this difference with different symbols.

Table S1. Summary and comparison of different approaches used in this work.

Approach	analytical	numerical	Fourier-based
Calculation domain	frequency	time	frequency and time
Access to medium dynamics	no	$\sigma_{ij}(z,t)$	no
Access to field dynamics	$\Omega_p(z,t)$	$\Omega_p(z,t)$, $\Omega_k(z,t)$	$\Omega_p(z,t)$
Group index evaluation	spectral components $n_g(\delta)$ [Eq. (4)]	index per pulse $n_g(\delta_p)$ [Eq. (5)]	index per pulse $n_g(\delta_p)$ [Eq. (5)]
Accurate for spectrally broad pulses	no	yes	yes
Calculation speed	high	low	moderate

where σ_p^{stat} is the stationary value of the proper coherences ($p =$ "ba" for the V-type while $p =$ "ab" for the Λ- and Ξ-type systems). We solve Eqs. (1) for the coherences $\sigma_{ba}^{\text{stat}}$ (or $\sigma_{ab}^{\text{stat}}$) with the assumption that the interaction with the control field and operation under the steady state provide that prior to switching on the probe field whole population resides in the state $|b\rangle$ in the Λ and Ξ configurations (as for the V-system see the discussion below) and other levels are empty. Next, we expand the stationary expression in the Taylor series with respect to the values of Ω_p, which we truncate at the terms that are linearly proportional. The truncation is valid for the probe field of the Rabi frequency much smaller than the spontaneous-emission rates, $\Omega_p \ll \gamma$. We find that the electric susceptibility for all three energy configurations is

$$\chi_{ba}^{V}(\delta) = \mathrm{i}\frac{N|d_{ab}|^2}{\hbar\varepsilon_0}\frac{\left(\frac{\gamma_{cc}^2}{4}+\delta_k^2+\Omega_k^2\right)-\left(-\mathrm{i}\delta_k+\frac{\gamma_{cc}}{2}\right)\frac{\Omega_k^2}{-\mathrm{i}(\delta-\delta_k)+\frac{\gamma_{bb}+\gamma_{cc}}{2}}}{\left(\frac{\gamma_{cc}^2}{4}+\delta_k^2+2\Omega_k^2\right)\left[-\mathrm{i}\delta+\frac{\gamma_{bb}}{2}+\frac{\Omega_k^2}{-\mathrm{i}(\delta-\delta_k)+\frac{\gamma_{bb}+\gamma_{cc}}{2}}\right]}+\mathscr{O}[\Omega_p^2], \tag{S24a}$$

$$\chi_{ab}^{\Lambda}(\delta) = \frac{N|d_{ab}|^2}{\hbar\varepsilon_0}\frac{\mathrm{i}}{-\mathrm{i}\delta+\frac{\gamma_{aa}}{2}+\frac{\Omega_k^2}{-\mathrm{i}(\delta-\delta_k)}}+\mathscr{O}[\Omega_p^2], \tag{S24b}$$

$$\chi_{ab}^{\Xi}(\delta) = \frac{N|d_{ab}|^2}{\hbar\varepsilon_0}\frac{\mathrm{i}}{-\mathrm{i}\delta+\frac{\gamma_{aa}}{2}+\frac{\Omega_k^2}{-\mathrm{i}(\delta+\delta_k)+\frac{\gamma_{cc}}{2}}}+\mathscr{O}[\Omega_p^2]. \tag{S24c}$$

In the main text, we present graphically the above equations in Fig. 2. Used parameters represent typical orders of magnitude in atomic alkali vapors. The concentration is set to $N = 6.6\cdot 10^9$ atoms/cm^3 (for the **Hartree atomic unit system**, $\hbar = a_0 = m_e = e = 1$ used in the numerical calculations, the concentration is 10^{-15} a.u.). The spontaneous emission rates (γ_{aa}, γ_{bb}, and γ_{cc}) are calculated for each configuration based on the arbitrarily selected values $\gamma'_{ba} = \gamma'_{ac} = \gamma' = 2\pi\cdot 1.6$ MHz ($2.5\cdot 10^{-10}$ a.u.), and $\gamma'_{bc} = 0$ used in Eq. (S3) (see Sec. S2 for interpretation). This gives $\gamma_{aa} = 4\gamma'$ for the Λ configuration, $\gamma_{bb} = \gamma_{cc} = 2\gamma'$ for the V configuration, and $\gamma_{aa} = \gamma_{cc} = 2\gamma'$ for the Ξ configuration. The control field is set to $\Omega_k = 55\gamma' = 2\pi\cdot 90.1$ MHz ($1.38\cdot 10^{-8}$ a.u.). The dipole moments are calculated from the Weisskopf-Wigner theorem[2]

$$|d_{ab}|^2 = \frac{3\pi\varepsilon_0\hbar c^3}{\omega_{ab}^3 n}\gamma_{ab}, \tag{S25}$$

where γ_{ab} is the optical-coherence (σ_{ab}) relaxation rate due to spontaneous emission, and n is the refractive index of the medium. Analogously, we can find the value of $|d_{ca}|^2$.

S5 Validation of approaches

Here, we provide the comparisons of the methods presented in Sec. 4 of the main text. A concise summary is presented in Table S1. We discuss the impact of the probe field on the electric susceptibility, the accuracy of the Fourier transformation method compared to the direct-propagation approach, and the validity of the monochromatic-wave approach. We use the parameters for rubidium atoms as presented in Sec. 5.1 of the main text.

S5.1 Electric susceptibility with probe field

In the main paper, we calculated signals using Eq. (3). In the equation, we neglect the higher-order corrections, e.g., the terms proportional to Ω_p^2, as they are small under our conditions. Here, instead of using the Taylor expansion and neglecting the higher-order terms, we assume large single-photon detuning δ_p so that the whole population remains in the ground state $|b\rangle$, $\sigma_{bb}^{\text{stat}} \approx 1$ (this is true in many physically relevant cases). In consequence, the populations of the upper states are negligible, $\sigma_{aa}^{\text{stat}} \approx \sigma_{cc}^{\text{stat}} \approx 0$, and no significant coherence can arise between the levels, $\sigma_{ac}^{\text{stat}} \approx 0$. Under such conditions, the electric susceptibility of the Ξ-type medium for the probe field is given by

$$\chi_{ab}(\delta_p) = \frac{N|d_{ab}|^2}{\hbar\varepsilon_0} \frac{\mathrm{i}}{-\mathrm{i}\delta_p + \frac{\gamma_{aa}}{2} + \frac{\Omega_k^2}{\underbrace{-\mathrm{i}(\delta_p+\delta_k) + \frac{\gamma_{cc}}{2}}_{\substack{\text{two-photon}\\\text{resonance}\\\text{term}}} + \underbrace{\frac{\Omega_p^2}{-\mathrm{i}\delta_k + \frac{\gamma_{aa}+\gamma_{cc}}{2}}}_{\substack{\text{probe-field}\\\text{correction}}}}}. \tag{S26}$$

Contrary to Eq. (3), this expression includes the probe field Ω_p in all orders, taking into account nonlinear corrections to the medium response. Rewriting the denominator in Eq. (S26), we get

$$-\mathrm{i}(\delta_p+\delta_k) + \frac{\gamma_{cc}}{2} + \frac{\Omega_p^2}{-\mathrm{i}\delta_k + \frac{\gamma_{aa}+\gamma_{cc}}{2}} = -\mathrm{i}\left[\delta_p + \underbrace{\delta_k\left(1 - \frac{\Omega_p^2}{\delta_k^2 + \left(\frac{\gamma_{aa}+\gamma_{cc}}{2}\right)^2}\right)}_{\delta_k^{\text{eff}}}\right] + \underbrace{\frac{\gamma_{cc}}{2} + \frac{\frac{\gamma_{aa}+\gamma_{cc}}{2}\Omega_p^2}{\delta_k^2 + \left(\frac{\gamma_{aa}+\gamma_{cc}}{2}\right)^2}}_{\gamma_{bc}^{\text{eff}}}, \tag{S27}$$

where the probe field acts as a correction to the detuning δ_k and moves the two-photon resonance towards the single-photon resonance (an effective detuning δ_k^{eff}). Additionally, the resonance is broadened by the probe field (the effective rate γ_{bc}^{eff}). It should be noted, however, that, for the investigated case of $\delta_k \gg \frac{\gamma_{aa}+\gamma_{cc}}{2}$, the peak shift is the dominant effect.

S5.2 Comparison of results for monochromatic waves

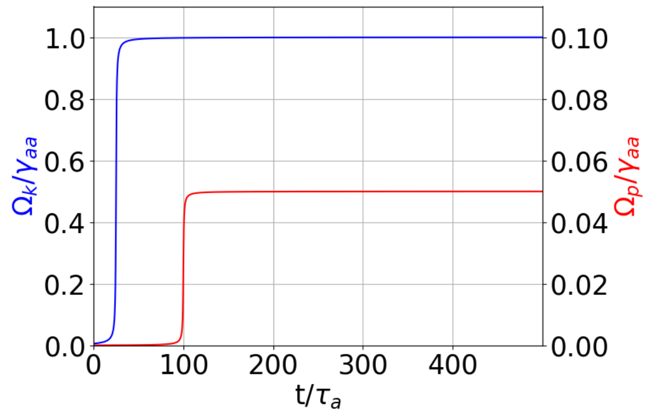

Figure S2. Probe- and control-field envelopes (respectively red and blue lines). Both shapes are presented right before entering the medium (probe for $z=0$ and control for $z=L$). For the probe field $t_m = -100/\gamma_{aa}$ while for the control field $t_m = -25/\gamma_{aa}$. For both pulses $a = 2\gamma_{aa}$.

We check the simplest possible case of monochromatic wave propagation in order to compare all three approaches introduced in Sec. 4 of the main paper.

Let us first note that considering the propagation of monochromatic waves is numerically ambiguous as the field needs to have a beginning and end. One may think of the step function as an approximation of a real-life realization of such a

problem, however, a fast switching of the field has numerical consequences and leads to unwanted oscillations known as the "switching-on effect". Instead, we use the *arctan* function, for which we can control the slope of a rising-field envelope. At the beginning of the medium ($z = 0$), the field can be described by

$$f(t) = \Omega \left[\frac{1}{\pi} \arctan(a(t + t_m)) + \frac{1}{2} \right], \tag{S28}$$

where Ω is the maximum amplitude of the field, a is the slope parameter given in units of [1/s], and t_m is the shift of the pulse in time. Additionally, as we are interested in the steady state, we switch on the control field first and wait until the dynamics of the medium stabilizes. Next, we turn on the probe field and again await the steady state. The envelopes of the representative control and probe fields are presented in Fig. S2. The control field propagates in the negative z-direction and the probe field propagates in the positive direction.

S5.2.1 Weak probe field

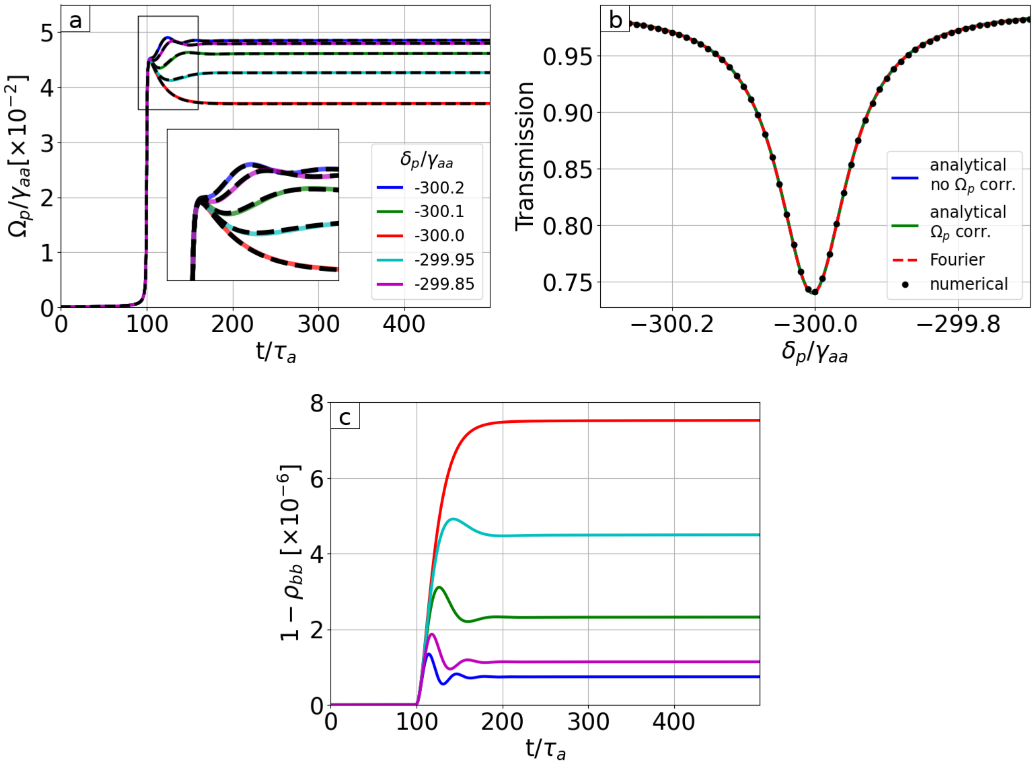

Figure S3. a) Shapes of the probe fields (solid colored lines) calculated numerically and obtained by the Fourier-based method (dashed black lines) at the end of the medium for different probe detunings. b) Absorption in spectral domain calculated based on: analytical [without (blue line) and with (green line) probe-field correction], Fourier (dashed red line), and numerical (black dots) approaches. c) Temporal change of population of the ground state $|b\rangle$.

For the consecutive calculations, we use parameters described in the main text as **Point 1**: control field $\Omega_k = \gamma_{aa}$, and detuning $\delta_k = 300\gamma_{aa}$. The probe field is also set to the same value $\Omega_p = 0.05\gamma_{aa}$. Once the steady state is reached, we measure the amplitude of the probe field at the end of the sample. All the calculations are done for a fixed detuning of the control field δ_k and for a range of detunings of the probe field δ_p around the two-photon resonance ($\delta_k + \delta_p = 0$). The resulting probe field envelopes at the end of the sample can be compared with those calculated with the Fourier-based method.

All the outcomes are presented in Fig. S3. First of all, in Fig. S3a we see pulse shapes for several detunings evaluated numerically (colored lines) and from the Fourier-based method (dashed lines). We can see a perfect agreement between these two methods. This suggests that the assumption $\sigma_{bb} \approx 1$ holds in that case. Indeed, Fig. S3c shows the transfer of population from state $|b\rangle$ for the point in the middle of the medium. The value is less than 0.001%.

In Fig. S3b we compare the transmission of the pulse obtained from the numerical calculations (black dots) with analytical results. We use the Lambert-Beer law

$$\Omega_p(L) = \Omega_p(0)\mathrm{e}^{-\frac{\omega_p}{2c}\,\mathrm{Im}(\chi_{ab})L}, \tag{S29}$$

where χ_{ab} is given by Eq. (S26). Since we intend to examine the importance of the probe-field correction, we plot transmission calculated with and without this correction (respectively, green "analytical Ω_p corr." and blue "analytical no Ω_p corr.". On top of that, we also plot the results from the Fourier-based method (dashed red). All the methods return indistinguishable results as all the curves are the same.

S5.2.2 Strong probe field

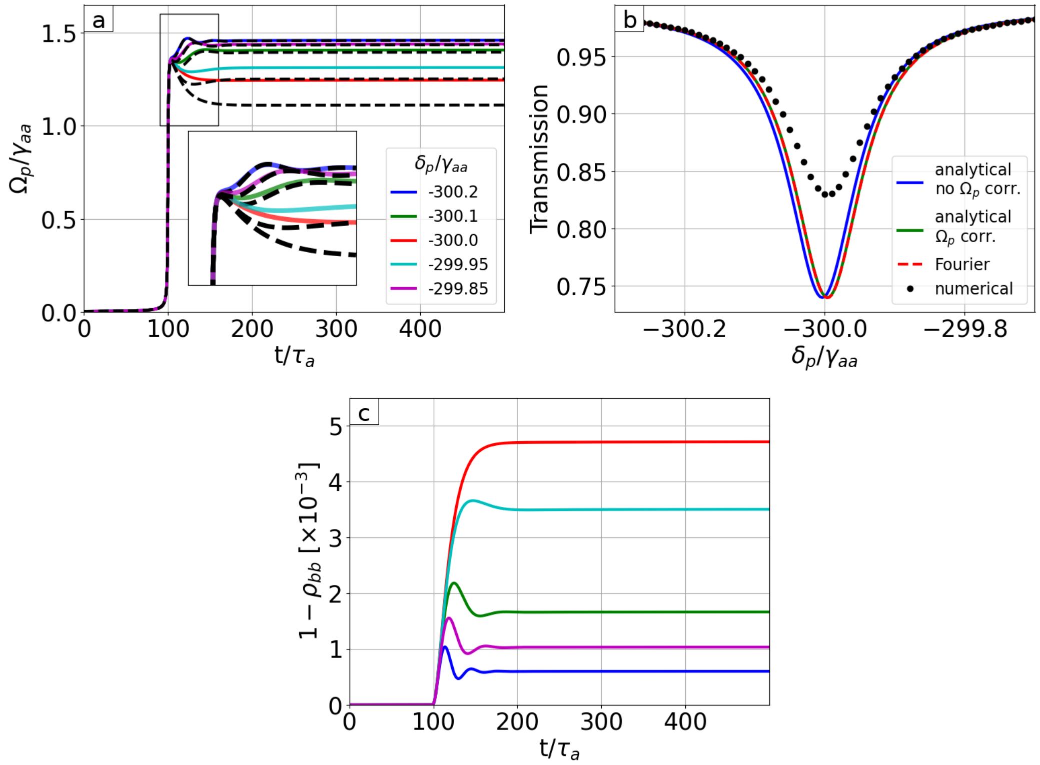

Figure S4. a) Pulse shapes at the end of the medium calculated numerically (solid lines) and determined using the Fourier approach (black dashed lines). The clear differences between the results of the two approaches indicate significant absorption of the control field along the process. b) Absorption in the spectral domain calculated using analytical [without (blue line) and with (green line) probe-field correction], Fourier (red line), and numerical (black dots) approaches. The resonance shift is evident and calculated correctly by incorporating the Ω_p-correction (the red plot is on top of the green one). c) Population of the ground state due to the action of the probe field, indicating the transfer of population to the excited state.

For a strong probe field, the nonlinear Ω_p-dependent correction in Eq. (S26) may modify the optical response of the medium. To investigate this, in the following, we discuss the test case of $\Omega_p = 1.5\gamma_{aa} > \Omega_k$ while all other parameters are the same as above. Note that the probe field is far-detuned with $\delta_p \approx -300\gamma_{aa}$.

As shown in Fig. S4a and S4b, for the strong probe field, the analytical and Fourier approaches are no longer valid near the resonance. This is due to the significant absorption of the control field, which modifies the properties of the medium. Near the resonance, the control field may be significantly absorbed, hence the medium response to the probe field is weaker. However, even for the case of the strong fields, the transfer of population to the excited states is negligible (less than 0.5%), as shown in Fig. S4c). From Fig. S4b, we can see that the probe-field correction is required at least for the proper description of the resonance position, as we can clearly see a resonance shift concerning the calculations neglecting the probe field.

We demonstrated that the assumption of the small probe field is valid unless the field intensity is not comparable to the

control field. However, even in the opposite case, the assumption of negligible population transfer holds but the absorption of the control field starts to be important significantly changing the probe absorption.

References

1. Tempel, D. G. & Aspuru-Guzik, A. Relaxation and dephasing in open quantum systems time-dependent density functional theory: Properties of exact functionals from an exactly-solvable model system. *Chem. Phys.* **391**, 130–142 (2011).

2. Scully, M. & Zubairy, M. *Quantum Optics, Sixth Printing* (Cambridge University Press Cambridge, 2008).

3. Breuer, H.-P. & Petruccione, F. *The theory of open quantum systems* (Oxford University Press, 2007).

4. Runge, C. Über die numerische auflösung von differentialgleichungen. *Math. Annalen* **46**, 167–178 (1895).

5. Lax, P. Systems of conservation laws. Tech. Rep., Los Alamos National Lab (1959).

6. LeVeque, R. J. *Numerical methods for conservation laws*, vol. 214 (Springer, 1992).
